# Host–microbiome archetypes differentiate infection from pathogen carriage in the human lower airway

**DOI:** 10.1038/s41467-026-71863-5

**Published:** 2026-04-13

**Authors:** Emily C. Lydon, Padmini Deosthale, Abigail Glascock, Hoang Van Phan, Christina M. Osborne, Matthew K. Leroue, Jawara Allen, Eran Mick, Brandie D. Wagner, Joseph L. DeRisi, Lilliam Ambroggio, Peter M. Mourani, Charles R. Langelier

**Affiliations:** 1 Division of Infectious Diseases, Department of Medicine, University of California, San Francisco, CA USA; 2 Chan Zuckerberg Biohub, San Francisco, CA USA; 3 Division of Pediatric Infectious Diseases, University of Pennsylvania, Perelman School of Medicine and Children’s Hospital of Philadelphia, Philadelphia, PA USA; 4 Division of Pediatric Anesthesiology and Critical Care Medicine, University of Pennsylvania, Perelman School of Medicine and Children’s Hospital of Philadelphia, Philadelphia, PA USA; 5 Department of Pediatrics, University of Colorado and Children’s Hospital Colorado, Aurora, CO USA; 6 Department of Biostatistics and Informatics, Colorado School of Public Health, University of Colorado, Aurora, CO USA; 7 Department of Biochemistry and Biophysics, University of California, San Francisco, CA USA; 8 Department of Pediatrics, University of Michigan, Ann Arbor, MI USA

**Keywords:** Microbiome, Molecular medicine, Infectious diseases, Metagenomics

## Abstract

Distinguishing lower respiratory tract infection (LRTI) from incidental pathogen carriage (IPC) is clinically challenging. The immunologic and microbial factors defining the states of LRTI and IPC are poorly understood. Here, we perform host-microbe metatranscriptomic profiling of tracheal aspirates from 326 mechanically ventilated children with clinically adjudicated LRTI (*n* = 207), IPC (*n* = 70), or non-infectious respiratory failure (*n* = 49). In the airway microbiome, LRTI shows reduced alpha diversity and taxonomic richness, while IPC displays greater bacterial abundance, enrichment in respiratory anaerobes, and increased metabolic activity. At the host level, patients with LRTI exhibit a distinct lower airway transcriptional signature of innate and adaptive immune activation compared to those with IPC, who have similar transcriptional profiles to uninfected controls. Mediation analyses suggest the airway microbiome influences the host response to pathogens. An integrated host-microbe metatranscriptomic classifier accurately discriminates LRTI from IPC and controls (AUC = 0.89, 95% confidence interval (CI) 0.85–0.92). The single gene *FABP4*, encoding a macrophage-associated lipid chaperone and recently described pneumonia biomarker, performs similarly when combined with alpha diversity; FABP4 protein alone achieves an AUC = 0.88 (95% CI 0.82–0.93). Together, our findings reveal distinct ecological and immunologic archetypes defining LRTI and IPC, and support data-driven, biology-informed LRTI diagnostics incorporating host and microbial features.

## Abstract (web summary)

Here, by integrating host gene expression and airway microbiome data, the authors distinguish true lower respiratory infection from incidental pathogen carriage, and identify biologically distinct patterns that improve diagnostic precision beyond pathogen detection alone

## Introduction

The upper and lower airways harbor robust microbial communities, which together represent the human respiratory microbiome^1^. While primarily comprised of commensal bacteria during states of health, it has been well established that respiratory pathobionts, or microbes with the potential to cause disease, can be incidentally carried within the airway microbiome without eliciting signs or symptoms of infection^2^. This phenomenon of colonization, or incidental pathogen carriage (IPC), which involves a dynamic relationship between pathobiont, microbiome, and host immune response, remains incompletely understood.

IPC frequently complicates the management of acute respiratory illness by confounding accurate lower respiratory tract infection (LRTI) diagnosis. For instance, in patients hospitalized for respiratory failure, the underlying cause often remains unclear for days, as both infectious and non-infectious conditions can present with overlapping clinical features. LRTI diagnostic tests that rely exclusively on pathogen detection cannot differentiate between true infection and IPC, nor discern the presence of a non-infectious etiology^3,4^. Early postmortem studies of direct lung tissue demonstrated poor concordance between lung culture and histologically confirmed pneumonia, underscoring the limitations of microbiology-based studies alone^5^. However, in contemporary clinical practice, detection of any potential pathogen, particularly in the setting of respiratory failure, can reflexively lead to an LRTI diagnosis, even in the absence of true infection^6,7^. This contributes to unnecessary antimicrobial use and missed opportunities to diagnose and treat alternative causes of respiratory failure, such as cardiac conditions or autoinflammatory diseases^8–10^.

While IPC occurs across the age spectrum, the incidence is highest in children^11–13^. For instance, an estimated 33–90% of children incidentally carry *Streptococcus pneumoniae* in the airway, compared to <5% of adults^14–17^. Similarly, *Moraxella catarrhalis* colonizes the nasopharynx in 30–100% of infants but only 1–5% of adults^18–20^. Viral IPC is also far more common among children; based on population surveillance studies, an estimated 25% of asymptomatic young children incidentally carry at least one viral pathogen, in contrast to only 2% of adults^21–23^.

The high baseline rates of respiratory IPC in children are further increased in the setting of hospitalization and critical illness. The physiological disturbances of critical illness, including disruption of epithelial barriers, immune dysregulation, and introduction of endotracheal tubes, reshapes the respiratory microenvironment, promoting shifts in microbial community composition and facilitating colonization by opportunistic pathogens^24,25^. Among patients who require mechanical ventilation for non-infectious indications, airway colonization with potentially pathogenic organisms occurs in nearly half within the first 24 h^26,27^.

Despite its clinical relevance and frequent occurrence, the host and microbial factors that distinguish IPC from LRTI remain incompletely understood. No studies have yet evaluated IPC in the context of the lower airway microbiome and host response, and tests capable of accurately distinguishing LRTI from IPC do not yet exist. To address these gaps, we studied a prospective cohort of critically ill children hospitalized for acute respiratory failure and performed metatranscriptomic RNA sequencing on lower respiratory samples to simultaneously profile both host and microbe. We identify striking host and microbial biosignatures that distinguish the two states and then leverage findings to build accurate diagnostic classifiers with the potential to advance acute respiratory illness management.

## Results

### Patient cohort, clinical adjudication, and pathogen detection

Critically ill children with all-cause respiratory failure requiring mechanical ventilation (*n* = 457) were prospectively enrolled at eight U.S. hospitals between 2/2015 and 12/2017 (Supplementary Fig. S1)^28–30^. Tracheal aspirate (TA) was obtained within 24 h of intubation and stored in an RNA stabilizing agent. High-quality RNA sequencing data capturing both the respiratory microbiome and host transcriptome were generated on 343 patients.

LRTI cases were identified by structured, retrospective clinical adjudication following intensive care unit (ICU) discharge performed by ≥2 physicians trained in critical care medicine or infectious diseases using the CDC/NHSN PNU1 criteria^31^. Adjudicators, who had access to all clinical data in the electronic health record and were blinded to metatranscriptomic results, identified 224 patients (65.3%) with LRTI. Alternative, non-infectious etiologies of respiratory failure were adjudicated in 119 (34.7%) patients, and included neurologic conditions, anatomic airway abnormalities, toxin exposures, cardiac conditions, trauma and autoimmune disease. To comprehensively identify respiratory pathogens in the lower airway, a combination of standard-of-care clinical microbiologic testing and respiratory metatranscriptomics was performed. Following this process, 207 patients received a clinical diagnosis of LRTI and had a respiratory pathogen detected (“LRTI” group). Of those with a clear non-infectious cause of respiratory failure, 70 had a pathogen detected (“IPC” group) and 49 patients did not (“CTRL” group). Seventeen patients with clinically adjudicated LRTI but negative microbiologic testing were excluded from the analysis.

We found no differences in sex, race, ethnicity, or comorbidities between groups (Table 1). Patients in the LRTI group had a younger median age of 0.6 years (interquartile range (IQR) 0.2–2.1), compared to 1.6 years (IQR 0.9–6.4) in the IPC group and 9.5 years (IQR 1.3–14.4) in the CTRL group, reflecting the typical epidemiologic differences in pediatric respiratory failure^4,32^. Ventilator days and ICU length of stay were slightly longer in LRTI versus IPC, though hospital length of stay was similar and mortality was lower. Antibiotic usage prior to intubation was similar across all groups, and notably, most patients received antibiotics during their hospital course (LRTI 99.0%, IPC 91.4%, CTRL 83.7%).

**Table 1:** Demographic and clinical characteristics of the LRTI, IPC, and CTRL groups

	LRTI (*n* = 207)	IPC (*n* = 70)	*P* value	CTRL (*n* = 49)
Female, *n* (%)	124 (59.9)	38 (54.3)	0.49	24 (49.0)
Male, *n* (%)	83 (40.1)	32 (45.7)		25 (51.0)
Age in years, median (IQR)	0.6 (0.2–2.1)	1.6 (0.9–6.4)	1.1e−05	9.5 (1.3–14.4)
Race, *n* (%)			0.53	
White	122 (58.9)	39 (55.7)		27 (55.1)
Black/African American	41 (19.8)	10 (14.3)		11 (22.4)
Asian	9 (4.3)	4 (5.7)		5 (10.2)
Native Hawaiian/Pacific Islander	1 (0.5)	2 (2.9)		0 (0.0)
American Indian/Alaska Native	4 (1.9)	1 (1.4)		0 (0.0)
Multi-racial	5 (2.4)	3 (4.3)		1 (2.0)
Unknown	25 (12.1)	11 (15.7)		5 (10.2)
Hispanic/Latino ethnicity, *n* (%)	41 (19.8)	18 (25.7)	0.38	3 (6.1)
Comorbidities, *n* (%)	86 (41.5)	37 (52.9)	0.13	22 (44.9)
Admission category, *n* (%)			4.4e−11	
Medical	206 (99.5)	52 (74.3)		29 (59.2)
Surgical	1 (0.5)	10 (14.3)		10 (20.4)
Trauma	0 (0.0)	8 (11.4)		10 (20.4)
Antibiotics prior to intubation, *n* (%)	72 (34.8)	17 (24.3)	0.14	17 (34.7)
Any antibiotic use, *n* (%)	205 (99.0)	64 (91.4)	0.004*	41 (83.7)
Ventilator days, median (IQR)	7.0 (5.0–9.0)	6.0 (4.0–7.0)	0.003*	6.0 (5.0–9.0)
ICU length of stay, median (IQR)	11.0 (8.0–16.0)	9.0 (7.0–12.0)	0.009*	10.0 (7.0–15.0)
Hospital length of stay, median (IQR)	16.0 (11.0–24.0)	17.0 (9.0–30.0)	0.73	23.0 (14.0–43.0)
Mortality, *n* (%)	6 (2.9)	10 (14.3)	0.001*	2 (4.1)

Race is indicated as “unknown” if patient declined or was unable to answer, if they selected “other” as an option, or if data were missing. *P* values compare LRTI and IPC groups. Fisher’s exact test used for categorical variables, and Wilcoxon rank-sum test used for continuous variables.

*IQR* interquartile range, *ICU* intensive care unit.

*Indicates statistically significant (*P* < 0.05).

**Fig. 1:**
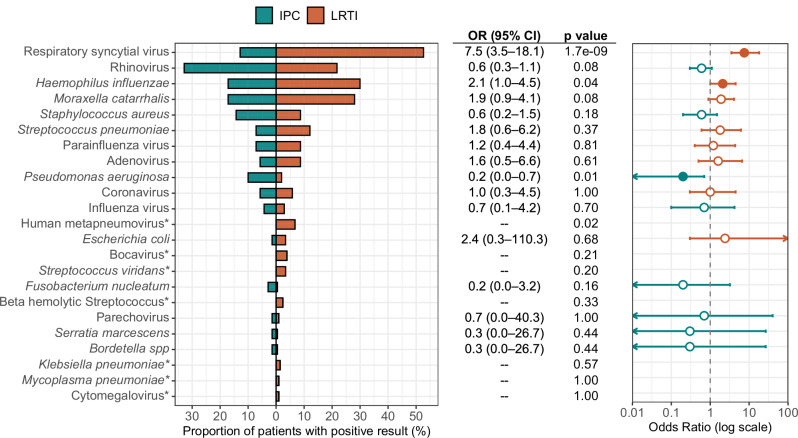
Distribution of pathogens among patients with clinically adjudicated LRTI or IPC. Bar plot demonstrating the proportion of participants in the LRTI (*n* = 207) and IPC (*n* = 70) groups with each detected pathogen as determined by combined clinical testing and metatranscriptomics. OR with 95% CI tabulated and plotted on the right. Filled circles represent statistically significant pathogens (*P* < 0.05). Asterisks indicate pathogens detected exclusively in one group, for which an OR could not be estimated. Arrows indicate where the lower or upper confidence interval exceeds the plotting area. Pathogens detected only once in the entire cohort were excluded from plotting. Statistical significance for between‑group differences was assessed with two-sided Fisher’s exact test with no adjustment for multiple comparisons. LRTI lower respiratory tract infection, IPC incidental pathogen carriage, OR odds ratio, CI confidence interval.

Respiratory syncytial virus (RSV) was the most common respiratory pathogen in the cohort, identified in 52.7% of the LRTI group and 12.9% of the IPC group (*P* < 0.001, Fisher’s exact test) (Fig. 1; detection stratified by clinical versus metatranscriptomics testing modality shown in Supplementary Fig. S2). Other pathogens that statistically differed in prevalence between groups included human metapneumovirus (LRTI 6.8%, IPC 0.0%, *P* = 0.02), *Haemophilus influenzae* (LRTI 30.0%, IPC 17.1%, *P* = 0.04), and *Pseudomonas aeruginosa* (LRTI 1.9%, IPC 10.0%, *P* = 0.01). Several other pathogens were identified at similar rates between groups, including rhinovirus, *Moraxella catarrhalis*, *Staphylococcus aureus*, and *Streptococcus pneumoniae*. Co-detection of both bacteria and viruses was common, comprising 59.4% of LRTI cases and 25.7% of IPC cases.

### The respiratory microbiome differs between LRTI, IPC, and controls

**Fig. 2:**
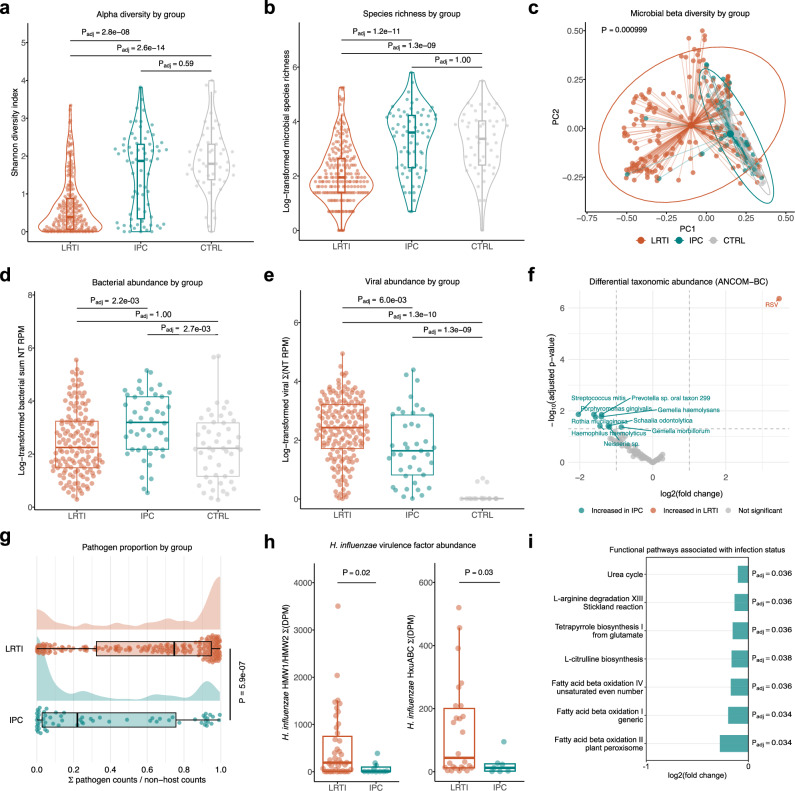
The lower airway microbiome differs between infection states. **a** SDI of the lung microbiome in patients with LRTI (*n* = 207, orange), IPC (*n* = 70, teal), or CTRL (*n* = 49, gray). *P* values were generated using a two-sided Wilcoxon rank-sum test and adjusted using the BH FDR algorithm. For all boxplots, box limits correspond to the IQR with the center line representing the median. The lower whisker extends to the smallest value within (1.5× IQR) below the first quartile, and the upper whisker extends to the largest value within (1.5× IQR) above the third quartile. **b** Species richness of the lung microbiome by group. Sample sizes and statistics are identical to panel (**a**). **c** Principal-coordinate analysis of Bray–Curtis distances demonstrating differences in community composition between groups; *P* value calculated by PERMANOVA. Ellipses represent the 95% confidence interval around the centroid of a given group. Sample sizes are identical to panels (**a**, **b**). **d** Lower airway total bacterial abundance measured in RPM (LRTI: *n* = 146, IPC: *n* = 46, CTRL: *n* = 49), restricted to patients with bacterial pathogens by composite microbiology and detectable bacterial reads passing background filtering. Statistical approaches are identical to panels (**a**, **b**). **e** Lower airway total viral abundance measured in RPM (LRTI: *n* = 177, IPC: *n* = 41, CTRL: *n* = 17), restricted to patients with viral pathogens by composite microbiology and detectable viral reads passing background filtering. Statistical approaches are identical to panels (**a**, **b**, **d**). **f** Differentially abundant taxa (LRTI: *n* = 207, IPC: *n* = 70) in the lower airway microbiome identified by ANCOM-BC. *P* values were generated using a two-sided Z-test applied to the test statistic (W) and corrected for multiple testing using the BH FDR algorithm. Taxa with adjusted *P* values < 0.05 are shown in color, corresponding to the group in which they are enriched. **g** Proportion of pathogen-assigned reads represented in the lower airway microbiome (LRTI: *n* = 204, IPC: *n* = 63), restricted to samples with detectable pathogen reads passing background filtering. *P* value was generated using a two-sided Wilcoxon rank-sum test. Density plots are shown above the boxplots to demonstrate the data structure. **h** Expression of *Haemophilus influenzae* virulence factors *HMW1/2* (LRTI: *n* = 43, IPC: *n* = 11) and *HxuABC* (LRTI: *n* = 26, IPC: *n* = 7), restricted to samples in which the respective virulence factors were detected. Statistical approaches are identical to panel (**g**). **i** Log₂-fold change in HUMAnN3-derived bacterial metabolic pathways found to be significantly different (*P*adj < 0.05) between groups. *P* values were generated using MaAsLin2 and corrected using the BH FDR correction. Sample sizes are identical to panel (**f**). All analyses were performed on independent biological samples from individual patients. SDI Shannon Diversity Index, LRTI lower respiratory tract infection, IPC incidental pathogen carriage, BH Benjamini–Hochberg, FDR false discovery rate, IQR interquartile range, RPM reads per million.

We first sought to compare the composition and function of the lung microbiome between LRTI and IPC, hypothesizing that both biologically relevant and diagnostically useful distinctions may exist. Alpha diversity demonstrated notable differences, with LRTI characterized by a lower Shannon Diversity Index (SDI) compared to either IPC (*P*_adj_ = 2.8e−8, Wilcoxon rank-sum test) or CTRL (*P*_adj_ = 2.6e−14). In contrast, SDI did not differ between IPC and CTRL groups (*P*_adj_ = 0.59) (Fig. 2a). Similarly, we found that community richness (total number of unique species in the lung microbiome) was significantly higher in both IPC and CTRL groups compared to those with LRTI (*P*_adj_ = 1.2e−11 and 1.3e−9, respectively) (Fig. 2b). Community composition also differed between LRTI, IPC, and CTRL groups based on the Bray–Curtis index (*P* < 0.001, PERMANOVA) (Fig. 2c). Despite these distinct microbiome archetypes, the microbe designated as the pathogen by our combined clinical-metagenomic framework was typically the most abundant microbe in the airway microbiome in both LRTI and IPC (Supplementary Fig. S3).

We next compared total bacterial abundance (measured in reads per million, RPM) between groups. Surprisingly, total bacterial abundance was highest in IPC compared to both LRTI (*P*_adj_ = 2.2e−3, Wilcoxon rank-sum test) and CTRL (*P*_adj_ = 2.7e−3) (Fig. 2d). In contrast, total viral RPM was highest in LRTI, while the IPC group exhibited an intermediate state with greater viral abundance compared to the CTRL group (*P*_adj_ = 1.3e−9) (Fig. 2e). Sensitivity analyses subsetting by viral-bacterial co-detection did not markedly change the observed relationships between LRTI, IPC, and CTRL groups (Supplementary Fig. S4).

We next examined pathogen abundance and found that rhinovirus RPM was significantly higher in LRTI compared to IPC (*P* = 0.047, Wilcoxon rank-sum test), while mean RSV abundance trended higher in LRTI, but the difference did not reach statistical significance (*P* = 0.060) (Supplementary Fig. S5). *H. influenzae* abundance did not differ between groups (*P* = 0.84), while *M. catarrhalis* RPM was unexpectedly higher in IPC compared to LRTI (*P* = 9.1e−3).

To further characterize the microbiome features of LRTI and IPC, we carried out differential taxonomic abundance analysis using ANCOM-BC^33^. While RSV was the only microbe with significantly greater abundance in LRTI, we found that IPC was enriched with classically commensal and anaerobic taxa including *Prevotella*, *Neisseria*, *Porphyromonas*, *Gemella*, and *Streptococcus* species (Fig. 2f, Supplementray Data 1). We subsequently evaluated pathogen burden, quantified as the proportion of non-human sequencing reads assigned to the implicated respiratory pathogen. A ratio near 1.0 indicates that the pathogen comprised nearly all microbial reads, while lower values (e.g., <0.2) indicate that the pathogen represented only a minority of the community. Although both groups demonstrated a wide range of pathogen burden, patients with LRTI were more likely to exhibit more pathogen-dominant communities, whereas in IPC, the pathogen more often comprised only a small fraction of the microbial community (63.4% versus 36.6% mean pathogen proportion, *P* = 5.9e−7, Wilcoxon rank-sum test) (Fig. 2g).

We hypothesized that virulence factor expression might differ between LRTI and IPC, and thus carried out an exploratory assessment using the MetaVF database^34^. We found that the expression of two *H. influenzae* virulence factors, *HMW1/2* and *HxuABC*, were higher in LRTI (*P* = 0.02 and *P* = 0.03, respectively, ANCOM-BC) (Fig. 2h, Supplementary Fig. S6, Supplementary Data 2). *HMW1/2* are adhesin proteins that facilitate *H. influenzae* adherence to the respiratory epithelium^35^, and *hxuABC* is a specialized ATP-binding cassette transporter for iron acquisition from the host^36^.

We additionally assessed microbiome functional differences by profiling metabolic pathways using HUMAnN^37^. We found that compared to LRTI, IPC was characterized by higher expression of metabolic pathways essential for fatty acid beta-oxidation, citrulline biosynthesis, and arginine degradation (Fig. 2i, Supplementary Data 3). Taken together, our findings suggested that IPC is characterized by a more diverse, taxonomically rich, abundant, and metabolically active respiratory microbiome compared to LRTI.

### Host airway transcriptional responses distinguish LRTI from IPC and controls

**Fig. 3:**
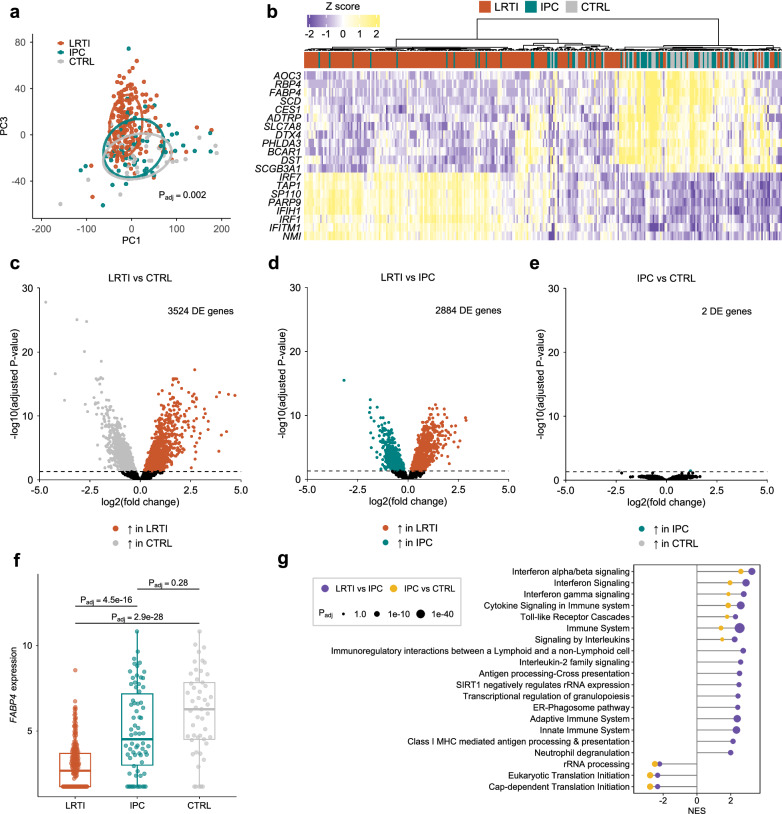
Lower airway host transcriptional responses distinguish LRTI from IPC and CTRL. **a** PCA of the lower respiratory tract host transcriptome. Adjusted *P* value calculated using two-sided PERMANOVA between LRTI and non-LRTI groups. **b** Heat map demonstrating hierarchical clustering of patients in each group (LRTI, IPC, CTRL) based on the top 20 DE genes between LRTI and CTRL groups. Color bar indicates normalized, Z-score scaled expression of each gene. **c** Volcano plot of DE genes between LRTI and CTRL. **d** Volcano plot highlighting DE genes between LRTI and IPC. **e** Volcano plot of DE results comparing IPC versus CTRL. DE was calculated using limma with moderated two-sided *t*-tests and BH adjusted *P* values < 0.05 colored. **f** Normalized *FABP4* expression across groups, with BH adjusted *P* values from the DE analyses. Box plots show the median (center line), interquartile range (box), and whiskers extending to the most extreme data points within 1.5× the interquartile range. **g** GSEA highlighting immune pathway enrichment in IPC compared to LRTI (purple) and IPC compared to CTRL (yellow). Top 20 DE pathways shown for the LRTI/IPC comparison, then overlaid with the IPC/CTRL comparison, showing only significant pathways (*P*_adj_ < 0.05). Point size scales inversely with *P*_adj_ value. GSEA used permutation-based enrichment statistics with BH adjustment. All analyses were performed on independent biological samples from individual patients. Sample sizes were LRTI *n* = 207, IPC *n* = 70, and CTRL *n* = 49. LRTI lower respiratory tract infection, IPC incidental pathogen carriage, CTRL control, PCA principal component analysis, DE differentially expressed or differential expression, BH Benjamini–Hochberg, GSEA gene set enrichment analysis.

We next tested the hypothesis that the pulmonary host response would differ between LRTI, IPC, and CTRL groups by evaluating the lower airway transcriptome. Principal component analysis demonstrated that LRTI was characterized by a distinct transcriptional signature compared to IPC or CTRL groups (*P*_adj_ = 0.002, PERMANOVA), but did not differ between IPC and CTRL (*P*_adj_ = 0.25) (Fig. 3a, Supplementary Fig. S7). This finding was underscored by hierarchical clustering of the top 20 most differentially expressed (DE) genes between LRTI and CTRL groups, which generally separated LRTI from non-LRTI cases, though several IPC cases clustered among LRTI cases (Fig. 3b). IPC and CTRL patients did not clearly separate based on hierarchical clustering.

To better understand host gene expression differences between the three groups at a more granular level, we performed pairwise differential expression analyses, adjusting for age and sex. We identified distinct host signatures that differentiated LRTI from IPC and CTRL groups, with 3517 and 2856 DE genes, respectively (Fig. 3c, Fig. 3d, Supplementary Data 4). In contrast, IPC and CTRL groups demonstrated minimal differences, with only 2 DE genes (Fig. 3e, Supplementary Data 4). Among the genes DE between LRTI and both CTRL and IPC groups, we noted that *FABP4*, which is expressed in macrophages and encodes a lipid chaperone that modulates leukotriene stability^38^, was a clear outlier in both fold change and statistical significance (Fig. 3f, Supplementary Fig. S8).

To characterize biological pathways encompassing the DE genes, we carried out gene set enrichment analyses (GSEA) (Fig. 3g, Supplementary Data 5). Canonical infection-related pathways, including interferon signaling, antigen presentation, adaptive immune signaling, and neutrophil degranulation, were all upregulated in LRTI versus IPC, as expected. However, we also noted that interferon signaling pathways were upregulated in IPC compared to CTRL patients, though to a lesser extent, suggesting that some, albeit subtle, induction of innate immune signaling occurs in the setting of pathogen presence in the airway.

**Fig. 4:**
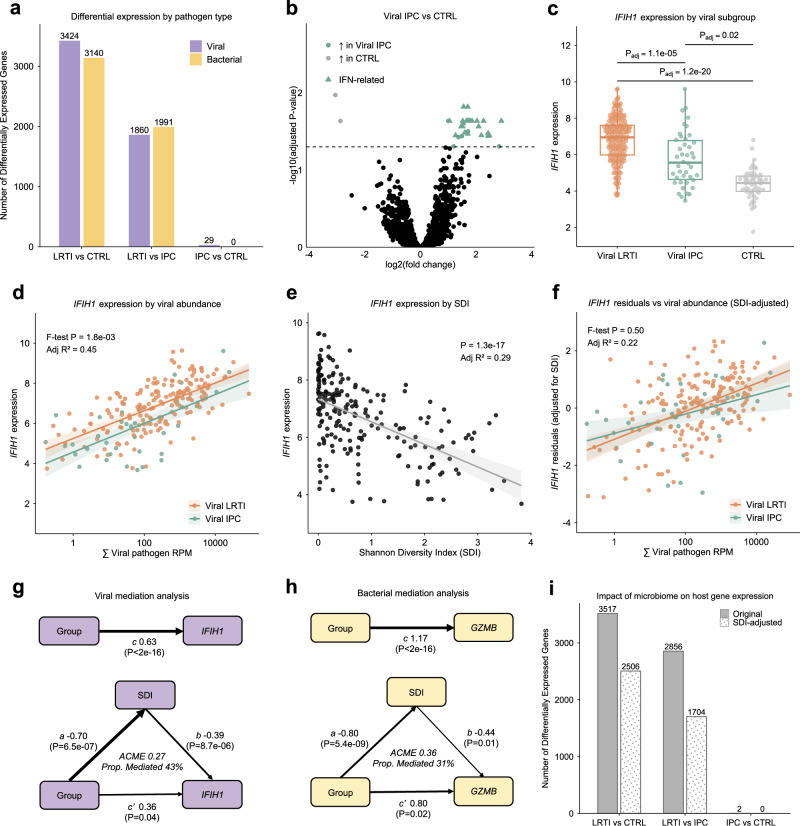
Innate immune activation differs between LRTI and IPC and is modulated by pathogen type, abundance, and microbiome diversity. **a** Number of DE genes between LRTI, IPC, and CTRL stratified by viral (purple) or bacterial (yellow) pathogen class. DE analyses were performed using limma with moderated two-sided t-tests and BH multiple-testing correction. **b** Volcano plot highlighting subtle ISG expression signature in viral IPC versus CTRL, derived from the same DE framework as in panel (**a**). **c** Boxplot of ISG *IFIH1* expression by group. BH adjusted *P* values from DE analyses shown. Box plots show the median (center line), interquartile range (box), and whiskers extending to the most extreme data points within 1.5× the interquartile range. **d** Linear regression of viral abundance (RPM) against *IFIH1* expression for viral LRTI and IPC. **e** Linear regression of SDI against *IFIH1* expression in viral subgroups. **f** Linear regression between viral abundance and *IFIH1* expression, after adjusting for SDI. Lines represent least-squares linear regression fits with shaded 95% CI. **g** Mediation analysis illustrating proportion of group effect mediated by SDI in viral cases. **h** Analogous mediation analysis for *GZMB* in bacterial cases. ACME and proportion mediated are shown. **i** Number of DE genes (*P*_adj_ < 0.05) between pairwise LRTI, IPC and CTRL comparisons, with and without adjustment for SDI, using limma with BH multiple-testing correction. All analyses were performed on independent biological samples from individual patients. Full cohort sample sizes were LRTI *n* = 207, IPC *n* = 70, and CTRL *n* = 49. For pathogen-stratified analyses, viral cases included patients with any virus detected by the composite microbiologic definition (viral LRTI *n* = 58, bacterial/viral LRTI *n* = 123, viral IPC *n* = 24, bacterial/viral IPC *n* = 18; total viral *n* = 223), and bacterial cases included patients with any bacteria detected by the composite microbiologic definition (bacterial LRTI *n* = 26, bacterial/viral LRTI *n* = 123, bacterial IPC *n* = 28, bacterial/viral IPC *n* = 18; total bacterial *n* = 195). LRTI lower respiratory tract infection, IPC incidental pathogen carriage, CTRL control, DE differentially expressed or differential expression, ISG interferon-stimulated gene, IFN interferon, BH Benjamini–Hochberg, RPM reads per million, SDI Shannon diversity index, CI confidence interval, ACME average causal mediation effect.

Given that interferon signaling is a central feature of the anti-viral host immune response, we hypothesized that the immunologic features of IPC may differ between viral and bacterial pathogens. To investigate this, we performed differential expression analyses within patients who had viral (*n* = 223) or bacterial (*n* = 195) pathogens detected. Aligning with our primary composite analysis, both viral and bacterial LRTI were characterized by distinct airway transcriptional signatures with respect to the CTRL group (3424 and 3140 DE genes, respectively) (Fig. 4a, Supplementary Data 6). Compared to IPC, viral and bacterial LRTI also exhibited distinct host signatures, although with fewer DE genes (1860 and 1991, respectively). Few transcriptomic differences were observed between IPC and CTRL groups, although a subtle signature of 29 DE genes, primarily interferon-stimulated genes (ISGs, e.g., *ISG15, IFIH1, OAS3*) characterized viral IPC (Fig. 4b). In contrast, there were zero DE genes between bacterial IPC and CTRL groups, suggesting fundamental differences in immune activation between patients incidentally carrying viral versus bacterial pathogens.

We next examined expression of individual genes classically associated with anti-viral and anti-bacterial defense. The viral subgroups demonstrated a gradation in ISG expression^39^, ranging from highest in viral LRTI to lowest in CTRL (Fig. 4c). Given the similar pattern with viral abundance in our microbiome analysis (Fig. 2e), we hypothesized that the differences in interferon signaling might simply be related to viral load differences between groups. A regression of ISG expression against viral RPM showed that ISG expression did positively correlate with interferon expression, as expected (adjusted R^2^ = 0.45), though interestingly, when stratified by group, IPC patients demonstrated a proportionally attenuated response compared to those with LRTI for any given viral load (*P* = 1.8e−3, linear regression interaction test for the ISG *IFIH1*) (Fig. 4d). When age was added as a covariate, this finding did not change (*P* = 2.0e−3), and other ISGs (*ISG15*, *IFI44*) exhibited the same pattern (Supplementary Fig. S9).

While these findings could be due entirely to intrinsic differences in innate immune response activation between individuals, we considered the possibility that the lung microbiome might modulate, at least to some extent, inflammatory gene expression in the setting of pathogen exposure. To investigate this, we evaluated the relationship between SDI and ISG expression and found that as lung microbiome diversity increased, ISG expression decreased (adjusted R^2^ = 0.29, linear regression *P* = 1.3e−17) (Fig. 4e). Furthermore, we found that after adjusting for SDI, between-group differences in the relationship between viral load and ISG expression disappeared (*P* = 0.50, linear regression interaction test), suggesting that the lung microbiome modulates anti-viral host responses (Fig. 4f). Mediation analysis suggested that lower SDI was independently associated with higher *IFIH1* expression, and that microbiome composition may partially mediate the relationship between viral pathogen presence and interferon signaling, explaining ~43% of the group effect on *IFIH1* expression (Fig. 4g).

We performed a parallel analysis focused on bacterial LRTI and IPC (Supplementary Fig. S10). In contrast to our observations with viral pathogens, the expression of canonical anti-bacterial innate immunity genes (*GZMB*^40^, *CD64*^41^, and *TLR1*^42^) remained relatively constant across a range of bacterial pathogen loads (e.g., for *GZMB*, adjusted R^2^ = 0.07). However, as with viral pathogens, the bacterial IPC group exhibited consistently lower innate immunity gene expression compared to the LRTI group (e.g., for *GZMB*, *P*_adj_ = 3.0e−04). Applying the same mediation analysis to *GZMB* demonstrated that lower lung microbiome alpha diversity was independently associated with higher innate immunity gene expression, explaining ~31% of the group effect on *GZMB* expression (Fig. 4h).

Lastly, we assessed the impact of adjusting for SDI in our original pairwise differential expression analyses and found that doing so markedly reduced the lower airway transcriptional differences between LRTI and IPC (Fig. 4i, Supplementray Data 7).

### Integration of host and microbial features enables accurate LRTI diagnosis

**Fig. 5:**
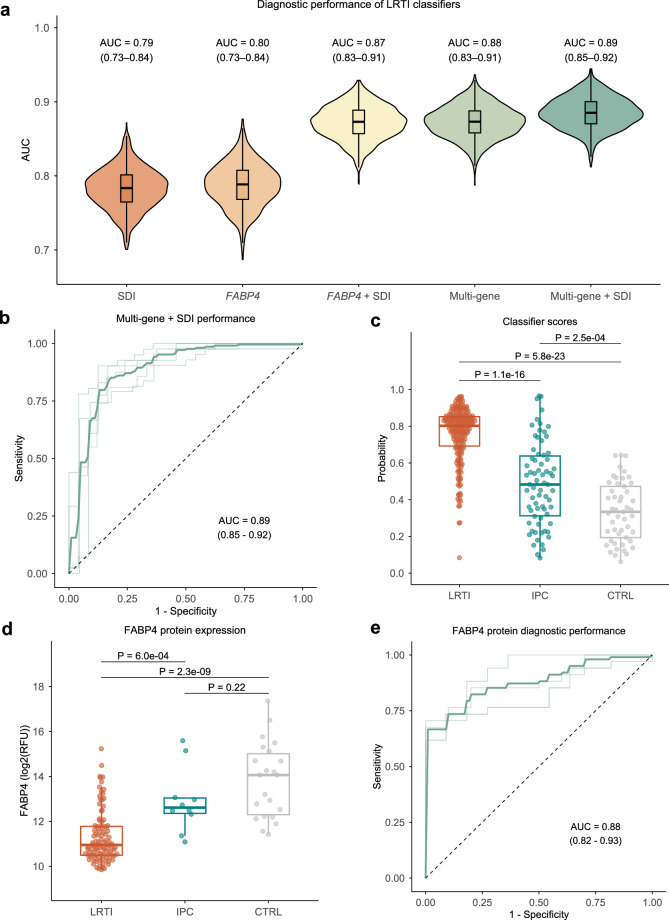
Integration of host and microbial features enables accurate diagnosis of LRTI and differentiation from IPC. **a** Comparative performance of classifiers for distinguishing LRTI from IPC or CTRL groups. Classifiers based on SDI, *FABP4, FABP4* + SDI, a multi-gene model derived from LASSO regularized regression of the host transcriptome, or a multi-gene model +SDI. AUC reflects mean across 5-fold cross-validation, and 95% CI estimated by bootstrapping. **b** ROC curve for multi-gene +SDI classifier. The thick line represents the mean ROC curve across 5 cross-validation folds, and thin lines represent ROC curves from individual folds. **c** Probability of LRTI based on classifier scores broken down by clinical group. *P* values were calculated using two-sided Wilcoxon rank-sum tests. Box plots show the median (center line), interquartile range (box), and whiskers extending to the most extreme data points within 1.5× the interquartile range. **d** Lower respiratory FABP4 protein concentration between LRTI, IPC and CTRL groups, with *P* values and box plots generated as in panel (**c**). **e** ROC curve for FABP4 protein biomarker. The thick line represents the mean ROC curve and thin lines represent ROC curves from individual cross-validation folds, as in panel (**b**). All analyses were performed on independent biological samples from individual patients. Transcriptomic classifier analyses used the full cohort (LRTI *n* = 207; IPC *n* = 70; CTRL *n* = 49), whereas FABP4 protein analyses were performed in a subset of patients with available protein measurements (LRTI *n* = 102; IPC *n* = 10; CTRL *n* = 22). LRTI lower respiratory tract infection, IPC incidental pathogen carriage, CTRL control, SDI Shannon diversity index, LASSO least absolutely shrinkage and selection operator, ROC receiver operating characteristic, AUC area under curve, CI confidence interval.

Having identified such distinct microbiome and host immune response differences between groups, we next sought to translate our findings into proof-of-concept diagnostic tests. Using LASSO regularized regression, we built diagnostic classifiers to distinguish true infection from the alternative clinically encountered states of IPC or non-infectious acute respiratory illness (Fig. 5a). Given prior work demonstrating the utility of *FABP4* as a pneumonia diagnostic biomarker^43,44^, we evaluated its performance alone or in combination with alpha diversity. Both *FABP4* and SDI performed well individually, although the combination achieved even better classification performance with an area under the receiver operator curve (AUC) of 0.87 (95% confidence interval (CI) 0.83–0.91) based on 5-fold cross validation (CV). A multi-gene host transcriptional classifier in combination with SDI performed comparably with an AUC of 0.89 (95% CI 0.85–0.92, Fig. 5b, Supplementary Table S1) and yielded classifier scores that effectively distinguished LRTI from patients in either the IPC or CTRL groups (Fig. 5c).

Considering that a single protein biomarker could have distinct practical utility as a clinical diagnostic, we tested whether protein levels of FABP4 could also effectively differentiate LRTI from IPC and CTRL groups in a subset of patients with FABP4 protein measurements from the lower airway (*n* = 134). Indeed, the LRTI group had markedly different levels of FABP4 compared to both IPC (*P* = 6.0e−4, Wilcoxon rank-sum test) and CTRL (*P* = 2.3e−9) groups (Fig. 5d). Consistent with our findings at the transcriptional level, no difference in FABP4 levels between IPC and CTRL groups was observed (Fig. 3f). Notably, we found that respiratory FABP4 alone performed as well as the integrated host/microbe metatranscriptomic classifier (AUC = 0.88, 95% CI 0.82–0.93) (Fig. 5e), suggesting promise as a clinical biomarker for both LRTI diagnosis and distinguishing LRTI from IPC.

## Discussion

Differentiating LRTI from IPC remains a frequent and unresolved challenge in the care of patients with acute respiratory illness. The resulting diagnostic uncertainty drives antimicrobial overuse and reflects a key gap in our understanding of host-microbe interactions in the lower airway. Here, we deploy metatranscriptomic profiling to holistically characterize the host and microbial features of LRTI and IPC, identifying distinct inflammatory signatures and respiratory microbiome ecology that distinguish these two states of pathobiont existence. Leveraging these findings, we develop host-microbe and practical single-biomarker LRTI diagnostic classifiers, offering a path toward more precise, biologically informed diagnostics.

Although the implicated pathogens were frequently the most abundant microbes in the lower airway microbiome in both patients with LRTI and IPC, microbiome alpha and beta diversity, and taxonomic richness were strikingly different. LRTI was marked by a collapse of alpha diversity, reflecting ecologic disruption that is characteristic of infection^28,45,46^, whereas IPC resembled uninfected controls, with a diverse and taxonomically rich community composition. Unexpectedly, total bacterial abundance was greater in IPC than in LRTI or controls, which may be explained by the enrichment of commensal taxa such as *Prevotella*, *Neisseria*, *Gemella*, and *Porphyromonas* and reflect a more resilient and balanced barrier microbiota. Many of the taxa enriched in IPC were oral-respiratory anaerobes, intersecting with the ongoing debate regarding the role of anaerobes as true pneumonia pathogens^47,48^. In this context, our findings suggest that enrichment of anaerobic taxa in the lower airway more often reflects innocuous carriage within a diverse microbial community rather than invasive infection, aligning with clinical pneumonia guidelines that generally do not recommend empiric anaerobic coverage^49^. Beyond taxonomic composition, the IPC state was further characterized by increased expression of diverse metabolic programs (e.g., energy production, fatty‑acid β‑oxidation, and amino‑acid biosynthesis) and higher virulence factor expression. Globally, these findings suggest that IPC is characterized by a more robust, diverse, and metabolically active microbiome, tolerant of carriage but resilient to pathogen invasion.

Host inflammatory gene expression in the lower airway also differed markedly between LRTI and IPC. We found that LRTI elicited a distinct transcriptional signature compared to either IPC or controls, comprised of thousands of DE genes related to innate and adaptive immune signaling. In contrast, the lower airway transcriptome of IPC largely resembled that of controls. Sensitivity analyses demonstrate that while no detectable transcriptional differences existed between bacterial IPC and controls, a subtle signal of interferon-stimulated genes distinguished viral IPC from controls. This pattern is consistent with prior reports of interferon activation during asymptomatic viral carriage^50,51^, but contrasts studies in neonates demonstrating that nasopharyngeal colonization with *M. catarrhalis*, *H. influenzae*, and *S. pneumoniae* correlates with mucosal immune shifts and the future development of asthma^52,53^. The discrepancy may be due to developmental stage (neonates were not included in our study and may be uniquely susceptible as their respiratory microbiomes are being established) or differences in upper versus lower respiratory tract biology^54^.

Regression analyses demonstrated that ISG expression is induced in a viral load-dependent manner, consistent with prior studies^55,56^. Intriguingly, however, ISG activation was consistently diminished in the setting of IPC across a range of viral loads. This suggested that IPC may be characterized by a global attenuation of the pathogen recognition-innate immune activation axis. Similar regression analyses involving bacterial cases did not demonstrate a dose-dependent relationship with respect to innate immune gene expression, perhaps reflecting fundamental differences in the coupling of bacterial antigens to the transcriptional activation of host innate immune responses. That said, we observed consistently higher expression of anti-bacterial immune genes (e.g., *GZMB*, *CD64*, *TLR1*) in LRTI compared to IPC, across a broad range of pathogen abundance, suggesting a fundamental set point difference between the two states, agnostic to pathogen class.

Our mediation analysis supports a role for the lung microbiome in moderating the intensity of inflammatory responses to potential invading pathogens. These findings, while proof-of-concept in nature, align with murine models in which microbiome disruption amplifies innate inflammatory responses and influenza-associated lung injury^57^. We estimated that microbiome factors explained ~43% of the relationship between group and inflammatory gene expression in viral cases, and ~31% in bacterial cases. While an important contribution, this suggests that other mediators (e.g., host genetics, epigenetic modifications, immune memory to related pathogens) primarily account for host responses differences between LRTI and IPC. Regardless, our findings underscore the complex, bi-directional relationship between host and microbe that determines whether a pathobiont will cause invasive disease or co-exist innocuously in a microbial community. We acknowledge that a full mediation analysis and determination of causality and directionality is not possible given the inherent limitations of an observational cohort^58^.

Distinguishing LRTI from IPC and non-infectious acute respiratory illnesses remains a clinical challenge and underscores the need for better diagnostic tests to guide antimicrobial therapy and patient care. We illustrate that simple host and microbial biomarkers can be used independently, or combined, to build clinically translatable diagnostic tests to address this need. For instance, *FABP4* in combination with SDI accurately distinguished children with proven LRTI from those with other causes of acute respiratory failure, including those with IPC, achieving an AUC of 0.87. As sequencing technology becomes more economical and clinically accessible, the feasibility and cost effectiveness of performing metatranscriptomic analyses will continue to improve^59–61^.

Inflammatory protein biomarkers (e.g., procalcitonin, C-reactive protein) are the most widely clinically available class of host-based infectious disease diagnostics, although they only have modest capability of diagnosing LRTI and have not been shown to effectively discriminate between LRTI and IPC^62,63^. Thus, we found it promising that FABP4 alone, when measured at the protein level, performed as well as our integrated host/microbe metatranscriptomic model (AUC = 0.88), highlighting the potential clinical utility of this single host biomarker for rapid and accurate diagnosis of infection in this cohort.

Our study has several strengths including the incorporation of both host and microbial data using metatranscriptomics, a multicenter design, and a large sample size. Our study also has limitations. First, LRTI status was defined using retrospective clinical adjudication performed following ICU discharge, an approach taken due to the absence of a true gold standard for pneumonia diagnosis^64^. Although adjudication was performed rigorously by ≥2 independent expert reviewers with consensus resolution, some misclassification is unavoidable and reflects the clinical uncertainty this study seeks to address. Second, we focused exclusively on children because overall they have a higher prevalence of IPC, thus it remains unknown whether our findings are generalizable to adults, or to individuals with less severe respiratory illnesses. The infection and IPC groups differed in age, although we adjusted for this in our analyses. While our study is the largest to date to examine biological differences between infection and IPC, our sample size did limit subanalyses at the individual pathogen level.

Given the inherent limitations of an observational cohort and cross-sectional study design, our mediation analyses should be considered proof-of-concept and will require validation in a more controlled experimental setting. Longitudinal sampling could help determine whether diversity collapse precedes, accompanies, or follows infection onset, a question that has been raised by early ICU studies using conventional microbiology that suggested abnormal bacterial dominance may precede clinical LRTI diagnosis by several days^65^, and supported by more recent sequencing-based studies demonstrating decreased community diversity prior to ventilator-associated pneumonia diagnosis^25^. Complementary experimental approaches, such as studies in xenobiotic mice, could more effectively establish causal relationships between microbiome and host inflammatory responses in the setting of pathobiont challenge. Our metatranscriptomic measurements reflect relative rather than absolute microbial abundance; future studies incorporating more quantitative or orthogonal approaches (e.g., DNA-based assays such as 16S rRNA gene quantification or external spike-in standards) will be useful to more precisely estimate microbial burden. Because this work was conducted in a pediatric cohort, future studies in adult populations will be important to determine whether the microbial and host response patterns that define LRTI and IPC are conserved across the age spectrum. Finally, future work is needed to evaluate whether our findings at the host and microbiome level generalize to the upper respiratory tract.

In sum, we find that LRTI and IPC are characterized by distinct biology with respect to both host and microbe, emphasizing that simply detecting a microbe with known pathogenicity in the respiratory tract is insufficient for clinical diagnosis of infection. It is not just the pathogen alone, but its dynamic relationship with the host immune response and airway microbiome, that determines disease. Our study provides fresh insight into the vexing and common challenge of interpreting positive respiratory tests in patients with acute respiratory illnesses and offers a new approach for improving LRTI diagnostic accuracy and limiting antimicrobial overuse.

## Methods

### Study cohort

We studied a prospective multicenter cohort of 457 critically ill children with acute respiratory illnesses requiring mechanical ventilation who were admitted to eight ICUs in the National Institute of Child Health and Human Development’s Collaborative Pediatric Care Research Network (CPCCRN) between February 2015 and December 2017^28–30^. Enrollment sites included: Children’s Hospital Colorado, Aurora, CO USA; University of California San Francisco, San Francisco, CA; Nationwide Children’s Hospital, Columbus, OH, USA; The Children’s Hospital of Philadelphia, Philadelphia, PA, USA; University of Pittsburgh, Pittsburgh, PA, USA; Children’s Hospital of Michigan, Detroit, MI, USA; University of California Los Angeles, Los Angeles, CA, USA; Children’s National Medical Center and George Washington School of Medicine and Health Sciences, Washington, DC, USA.

Children aged 31 days to 17 years who were expected to require mechanical ventilation for at least 72 h and had TA sampling performed within 24 h of intubation were approached for enrollment. Exclusion criteria included TA sample collection >24 h after intubation, presence of a tracheostomy tube, any condition in which deep tracheal suctioning was contraindicated, prior mechanical ventilation during the hospitalization, goals of care dictating a do not resuscitate order and/or a request for limited support, or previous enrollment in the study.

Eligible patients were identified, and their guardians were approached for consent by clinical research coordinator staff as soon as possible following intubation. Written informed consent for study participation was obtained from legal guardians. An initial waiver of consent was granted for TA samples to be obtained from standard of care suctioning of the endotracheal tube and stored until the parents or other legal guardians could be approached for informed consent. Samples from unconsented subjects were subsequently destroyed. The study was approved by the University of Utah central IRB #00088656.

### Adjudication of infection status and definition of subgroups

Adjudication of LRTI status was carried out retrospectively by study-site clinicians with access to all clinical, laboratory, microbiology, and radiology data available up to the end of admission, without knowledge of metatranscriptomic sequencing results. Each patient was reviewed independently by two adjudicators with expertise in pediatric infectious disease and/or critical care to determine the presence or absence of clinical LRTI; disagreements were discussed and resolved by a panel. For this study, patients were classified into three groups: 1) LRTI if they were clinically adjudicated as having LRTI and had supportive microbiology, 2) IPC if they were clinically adjudicated as not having LRTI but had positive microbiology, and 3) CTRL if they were clinically adjudicated as not having LRTI and had negative microbiology. Microbiology included standard-of-care clinical microbiology (multiplex polymerase chain reaction (PCR) and semiquantitative bacterial respiratory cultures) and/or metagenomic detection for pathogenic bacteria and viruses (implementing a validated, rules-based computational model, described in detail below)^28,29,45^. Additional details regarding clinical adjudication and interpretation of upper versus lower respiratory tract microbe detection can be found in the Supplementary Methods.

### RNA sequencing

TA collected within 24 h of intubation was mixed equi-volume with DNA/RNA shield (Zymo Research, Cat. No R1100) and stored at −80 °C. Following bead-bashing, samples underwent extraction using the Qiagen AllPrep Kit (Qiagen, Cat. No R2145), followed by DNAse treatment. All sample handling and RNA extractions were performed using a rigorous ultra-clean protocol designed for low biomass specimens, including use of personal protective equipment, RNase-decontaminated work surfaces and instruments, strict workflow separation, and processing of water-only controls in parallel to monitor and account for background contamination. Sequencing libraries were prepared from purified RNA using the NEBNext Ultra II Library Prep Kit (New England Biolabs, Cat. No E7770L) and dual index barcodes. Human ribosomal RNA depletion was carried out prior to library amplification and pooling using the Cas9-based Depletion of Abundant Sequences by Hybridization (DASH) method^66^. Libraries underwent 150-base pair paired-end sequencing on an Illumina NovaSeq 6000 sequencer.

### Measurement of FABP4 protein levels

FABP4 was measured from TA specimens collected within 24 h of intubation using the SomaScan 7k assay (SomaLogic)^67–69^ in a subset of this cohort. Following collection, TA specimens underwent centrifugation at 4 °C at 15,000 × *g* for 5 min, subsequently the supernatant was frozen at −80 °C within 30 minutes.

### Taxonomic mapping from RNA-seq data

We employed the CZ ID Illumina mNGS pipeline (v7.1) for taxonomic mapping of microbial sequence data^70,71^. This incorporates initial removal of human reads using Kallisto^72^, adapter sequence trimming with fastp^73^, filtering low quality and low complexity reads using PriceSeq^74^ and the Lempel–Ziv–Welch algorithm, respectively, and a final scrub of any residual human reads using Bowtie2^75^. Taxonomic classification was then performed on both short reads and assembled contigs using the NCBI nucleotide (NT) and nonredundant (NR) databases. Background and batch correction was performed on species level taxon matrices (see below).

### Identification and mitigation of background contaminants

Thirty-four negative water controls were processed and sequenced alongside the patient samples to enable characterization and subtraction of background contamination (Supplementary Data 8). A previously developed negative binomial model^55^ was used to model the distribution of reads of microbial taxa in the negative controls. Mean and dispersion parameters were fit to the data and estimates of the mean were generated for each batch:taxon pair. A single dispersion parameter was generated across all taxa using the MASS package (R, v7.3.58.1). *P* values were adjusted for multiple testing using the Benjamini–Hochberg False Discovery Rate method. Microbial taxa that were present at a significantly higher average abundance in participant samples than in negative controls (*P*_adj_ < 0.05) were retained for downstream analyses. Microbial taxa were included in downstream analysis if they met these criteria: (1) ≥1 hit to the NT database (2) ≥1 hit to the NR database (3) a minimum alignment length of 70 bases to the NT database.

### Clinical detection of respiratory pathogens

Standard-of-care clinical respiratory microbiologic testing was performed at the discretion of the treating clinicians at each study site. Diagnostics included nasopharyngeal swab respiratory pathogen testing by multiplex PCR and/or TA bacterial semiquantitative cultures. Clinical diagnostic tests on samples obtained within 48 h of intubation were included in the analyses. Microbes reported by the clinical laboratory as representing laboratory, skin, or environmental contaminants, or reported as mixed upper respiratory flora were excluded.

### Detection of respiratory pathogens by metatranscriptomics

For bacterial taxa that remained after background filtering, we applied an established rules-based model (RBM) to identify potential respiratory pathogens. In prior studies, the RBM identified 82–96% of clinically-confirmed lower respiratory pathogens compared to standard of care clinical diagnostics, and permitted detection of otherwise missed potential pathogens in >50% of patients with clinically adjudicated LRTI but negative standard testing^28,29,45^. The RBM operates by first retaining the most abundant species from each mapped genus, and any lower-abundance species within that genus with known pathogenicity in the respiratory tract based on a curated reference list from epidemiologic surveillance studies^76–79^. Species were then ranked by abundance (reads per million values aligned to NT database, sum NT RPM), limiting to the top 15. The largest drop in abundance among this ranked list was identified, and any species above the largest drop in abundance with known ability to cause LRTI as a potential pathogen were counted as bacterial hits. Viruses detected with an abundance >0.1 RPM with established human respiratory pathogenicity were subsequently identified.

### Microbial abundance calculations

Microbial abundance/load was approximated for each sample by calculating the sum NT RPM. Statistical comparison of sum NT RPM across groups was performed using the wilcox_test() function (rstatix v0.7.2). Resulting *P* values were adjusted using the Benjamini–Hochberg False Discovery Rate algorithm via the p.adjust() function of the stats package. Generalized linear modeling of these relationships was performed using the glm() function of the stats package, specifying a Gaussian distribution and identity link function, and adjusted for both sex and age. These methods were applied to the entire microbial profile as well as subsets of the profile (e.g., bacterial, viral) based on NCBI lineage data. Differences in abundance (sum NT RPM) of individual species of interest between groups were also performed using this approach.

### Microbiome diversity analyses

Alpha diversity (Shannon Diversity Index, or SDI) was calculated using the diversity() function of the vegan package (v2.7-1). Beta diversity (Bray–Curtis dissimilarity) was calculated using the functions vegdist(), betadisper(), permutest() and adonis2() of the vegan package. Principal Coordinate Analysis (PCoA) was performed using the cmdscale() function of the stats package.

### Differential microbial abundance analysis

Differential abundance analysis was performed using the ANCOM-BC package^33^ (v2.8.1) using a library filter of 0, prevalence filter of 10%, alpha level of 0.05 and a pseudo-count of 1. The analysis was adjusted for age and sex, and *P* values were adjusted for multiple testing using the Benjamini–Hochberg correction.

### Virulence factor screening

Virulence factors were identified from transcriptomic data using the MetaVF toolkit^34^, its associated virulence factor database VFDB2.0, and BLAST (v2.16.0). Called virulence factors with an associated e-value less than 1e−10 were retained for downstream analysis. Differentially expressed virulence factors were identified using ANCOM-BC^33^ with a library filter of 0, prevalence filter of 5%, alpha level of 0.05 and a pseudo-count of 1.

### Generation of host gene counts

RNA-seq reads were pseudoaligned using Kallisto^72^ against an index consisting of all transcripts associated with human protein-coding genes (GRCh38-based). We excluded samples with less than one million exon counts. Gene-level counts were generated using the tximport package, with the scaled TPM method (v1.28, Bioconductor 3.18)^80^. Genes were retained for subsequent analysis if they had at least 10 counts in at least 20% of the samples in the cohort. The gene counts table underwent variance-stabilizing transformation (VST) using the R package DESeq2 (v1.50.2)^81^, and VST-transformed counts were used in the principal component analysis, hierarchical clustering, assessment of individual genes, and classifier development.

### Principal component analysis

Principal component analysis (PCA) was performed on the complete gene expression matrix using the prcomp() function of the stats package. For data visualization, we plotted PC1 versus PC3, which provided the greatest apparent separation in two dimensions, and ellipses depict 68% confidence regions around group centroids. PC1 versus PC2 and PC2 versus PC3 are shown in the supplement. To formally assess group separation, we ran pairwise PERMANOVA (adonis2, vegan package v2.7-1) on Euclidean distances computed from the full expression matrix (i.e., testing differences in group centroids). *P* values from the pairwise contrasts were adjusted by the Benjamini–Hochberg method, with *P*_adj_ < 0.05 considered significant.

### Heat map and hierarchical clustering

For display, we selected the top 20 most significantly differentially expressed genes based in the LRTI and CTRL comparison, based on *P*_adj_ (see below for DE methods). Each gene was standardized across samples (z-score; mean = 0, SD = 1). Genes were ordered by unsupervised hierarchical clustering using Euclidean distance and complete linkage (ComplexHeatmap v2.26.1 defaults). Samples were clustered using correlation distance with Ward.D2 to emphasize similarity of gene expression profiles.

### Differential expression and gene set enrichment analyses

DE analyses were performed with the R package limma-voom (v3.66.0) on raw gene-level counts^82^. The design matrix included age and sex as covariates, and where noted, Shannon Diversity Index (SDI). Counts were transformed with voom (mean-variance modeling with precision weights) and quantile normalized across samples. Gene-wise statistics used empirical-Bayes moderated two-sided *t*-tests; multiple testing was accounted for by Benjamini–Hochberg with *P*_adj_ < 0.05 considered significant. Where individual genes are displayed (e.g., boxplots for *FABP4*, *IFIH1*, and *GZMB*), the adjusted *P* values from the respective limma DE analyses are displayed. For pathway analysis, we performed pre-ranked gene set enrichment analysis (GSEA) using ReactomePA (v1.54.0) on Reactome gene sets with a minimum pathway size of 10 genes and a maximum size of 1500 genes^83^. All genes from each limma DE comparison, ranked by the limma t-statistic, were included as input. For visualization, we displayed the top 20 DE pathways between LRTI and IPC (all statistically significant with *P*_adj_ < 0.05), then overlaid results from the IPC and CTRL comparison, only displaying the significant pathways.

### Regression and mediation analyses

For both viral and bacterial subgroup analyses (run in parallel with identical pipeline), we modeled gene expression using linear regression with log_10_ pathogen abundance (sum NT RPM) and group (LRTI versus IPC) as predictors, including an interaction term (gene expression ~ log_10_ pathogen abundance * group). Group differences were evaluated using a global F-test comparing this model to the null model (gene expression ~ log_10_ pathogen abundance), and sensitivity models additionally adjusted for age. For visualization, raw data points are plotted alongside model-predicted regression lines. The same structure was applied for microbiome diversity (gene expression ~ SDI * group). SDI-adjusted associations were visualized by plotting residuals from the gene expression ~SDI model against pathogen abundance with group-specific linear fits. To evaluate modulation of innate host gene expression by diversity, we used the mediation package with SDI as the mediator and included pathogen abundance as a covariate in both models (mediator: SDI ~ group + log10 pathogen abundance; outcome: expression ~ group + SDI + log10 pathogen abundance) and calculated average causal mediated effect (ACME) and average direct effect (ADE) with 1000 simulations using the mediation package (v4.5.1)^84^. The mediation models used simpler additive models to maintain the interpretability of the effect estimates and because the interaction terms were not significant in any of the regression models. Viral outcomes focused on interferon-stimulated genes (*IFIH1*, *ISG15*, *IFI44*) and bacterial outcomes on canonical anti-bacterial defense genes (*GZMB*, *CD64*, *TLR1*).

### Classifier development

Binary classifiers were developed to distinguish LRTI from non-LRTI (IPC + CTRL combined). We performed stratified five-fold cross-validation (same folds re-used across the different models, with a minimum IPC and CTRL counts to keep each fold balanced) and generated out-of-fold predictions for performance assessment. Single-feature models (i.e. *FABP4* gene, FABP4 protein, SDI) used logistic regression, as did *FABP4* + SDI. Multi-gene models used LASSO logistic regression on all genes (glmnet v4.1-10), with the regularization parameter lambda selected by internal cross-validation^85^. Non-zero coefficients selected by the LASSO model are provided in Supplementary Table S1. For performance metrics, the reported AUC reflects the mean AUC of each of the five folds computed with the pROC package (v1.19.0.1)^86^, and the confidence intervals were obtained by bootstrapping the out-of-fold predictions with 1000 resamples.

### Statistics and reproducibility

No statistical method was used to predetermine sample size; sample size was determined by the number of eligible participants enrolled in the parent cohort and for whom high-quality sequencing data were available. Sample exclusions occurred only for eligibility or quality control criteria, as described in the study flow diagram (Supplementary Fig. S1). No additional data were excluded from the analyses. The study was observational and experiments were not randomized. Clinical adjudication of infection status was performed retrospectively by two independent physician reviewers who were blinded to metatranscriptomic sequencing results, with disagreements resolved by consensus review. Statistical analyses were conducted in R v4.4.2 and v4.5.0. Differential gene expression analyses used limma-voom with empirical Bayes moderated two-sided *t*-tests and Benjamini–Hochberg correction for multiple testing. Group comparisons used Wilcoxon rank-sum or Fisher’s exact tests as appropriate. Classifier development used logistic regression or LASSO-regularized logistic regression with stratified five-fold cross-validation. Two-sided tests were used throughout and adjusted *P* values < 0.05 were considered statistically significant.

### Reporting summary

Further information on research design is available in the Nature Portfolio Reporting Summary linked to this article.

## Supplementary information

Supplementary Information

Description of Additional Supplementary Files

Supplementary Data 1

Supplementary Data 2

Supplementary Data 3

Supplementary Data 4

Supplementary Data 5

Supplementary Data 6

Supplementary Data 7

Supplementary Data 8

Reporting Summary

Transparent Peer Review file

## Source data

Source Data

## Data Availability

FASTQ files containing the microbial sequencing reads following subtraction of reads aligning to the human genome have been deposited in the NCBI Sequence Read Archive under BioProject accession PRJNA748764. Processed host gene counts, microbial taxon counts, and deidentified clinical metadata are available in the GitHub repository associated with this work: https://github.com/infectiousdisease-langelier-lab/Incidental_pathogen_carriage (10.5281/zenodo.19078486)^87^. Source data for each of the figures is provided in the Supplementary information. Source data are provided with this paper.

## Code availability

The code used for data processing, statistical analyses, and figure generation is publicly available at: https://github.com/infectiousdisease-langelier-lab/Incidental_pathogen_carriage (10.5281/zenodo.19078486)^87^.
