# Supplementary material for: Host–microbiome archetypes differentiate infection from pathogen carriage in the human lower airway
Source: Nat Commun. 2026 Apr 13;17:5149. doi: 10.1038/s41467-026-71863-5 (PMC13250161; doi:10.1038/s41467-026-71863-5)
Supplement: Supplementary file 1 — Supplementary Information [file 41467_2026_71863_MOESM1_ESM.pdf]

SUPPLEMENTARY MATERIAL

Host–Microbiome Archetypes Differentiate Infection from Pathogen Carriage in the Human Lower Airway

1. SUPPLEMENTARY FIGURES
2. SUPPLEMENTARY TABLES
3. SUPPLEMENTARY METHODS

1. SUPPLEMENTARY FIGURES

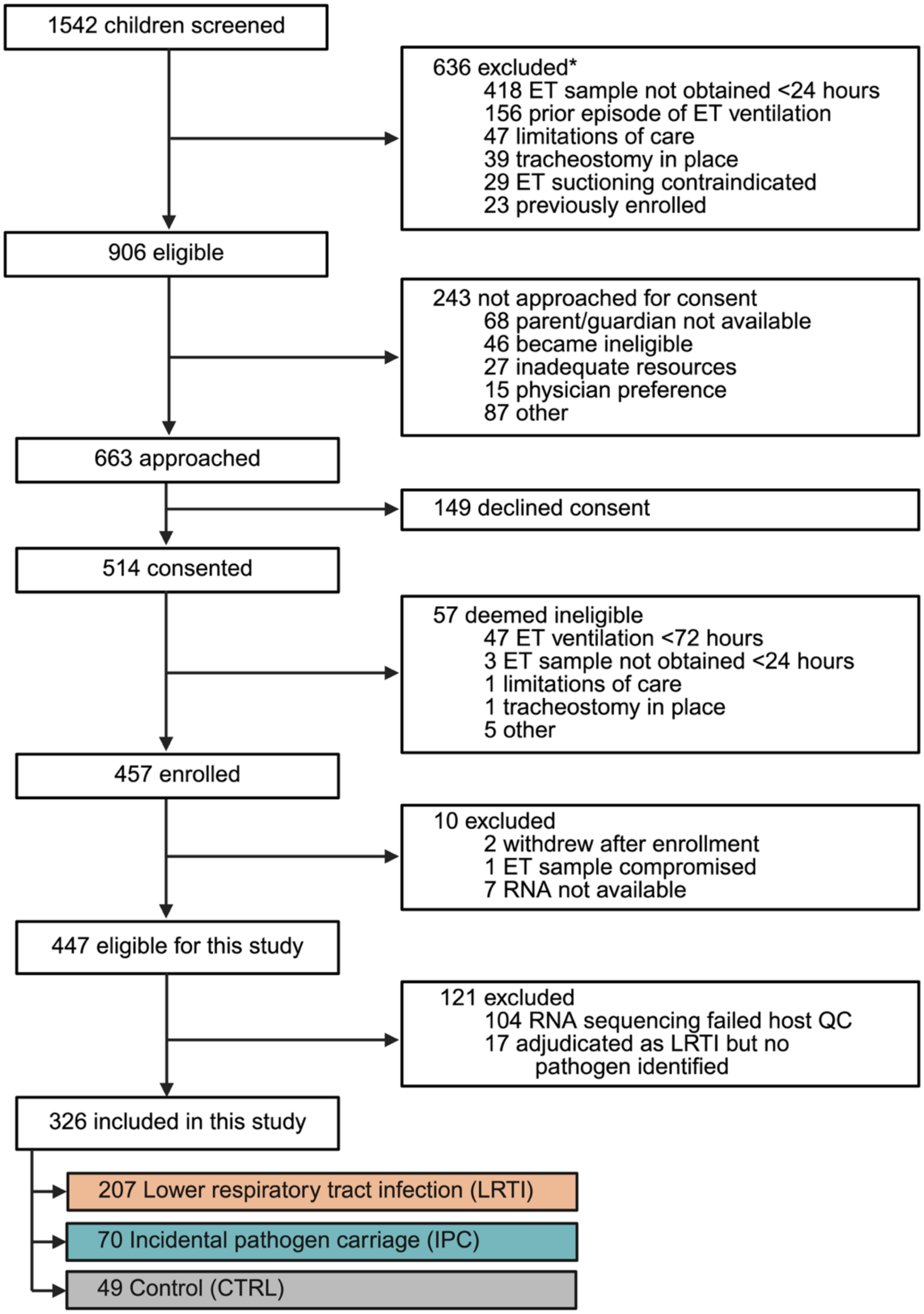

Figure S1. Study flow diagram. Asterisk indicates that some children had multiple reasons for exclusion. Flow diagram of patient enrollment, exclusions, and final cohort composition. Asterisk indicates that some children had multiple reasons for exclusion. Final cohort included patients with lower respiratory tract infection (LRTI, n=207), incidental pathogen carriage (IPC, n=70), and controls (CTRL, n=49). Abbreviations: ET, endotracheal tube; QC, quality control.

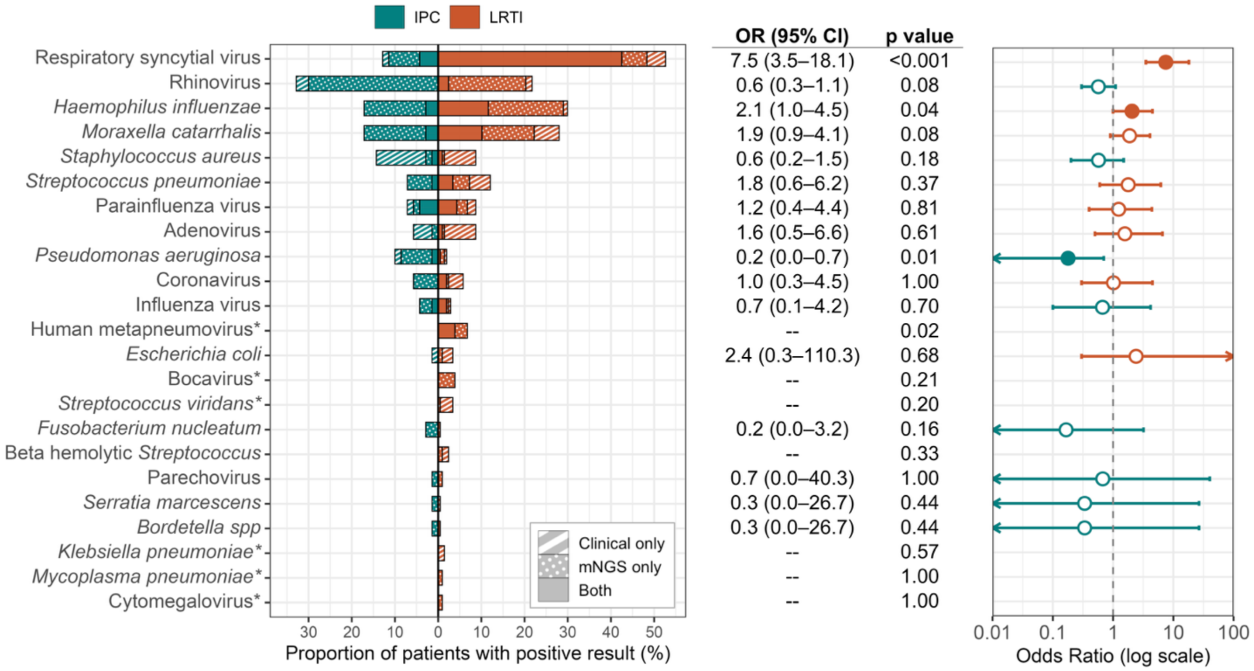

Figure S2. Distribution of pathogens stratified by detection modality in patients with clinically adjudicated LRTI or IPC. Bar plot demonstrating the proportion of participants in the LRTI (n=207) and IPC (n=70) groups with each detected pathogen as determined by combined clinical testing and metatranscriptomics and stratified by detection modality (clinical testing, metatranscriptomics, or both). Bar pattern denotes detection modality. OR with 95% CI tabulated and plotted on the right. Filled circles represent statistically significant pathogens (P<0.05). Asterisks indicate pathogens detected exclusively in one group, for which an OR could not be estimated. Arrows indicate where the lower or upper confidence interval exceeds the plotting area. Pathogens detected only once in the entire cohort were excluded from plotting. Statistical significance for between-group differences was assessed with two-sided Fisher's exact test with no adjustment for multiple comparisons. Abbreviations: LRTI, lower respiratory tract infection; IPC, incidental pathogen carriage; mNGS, metagenomic next generation sequencing; OR, odds ratio; CI, confidence interval.

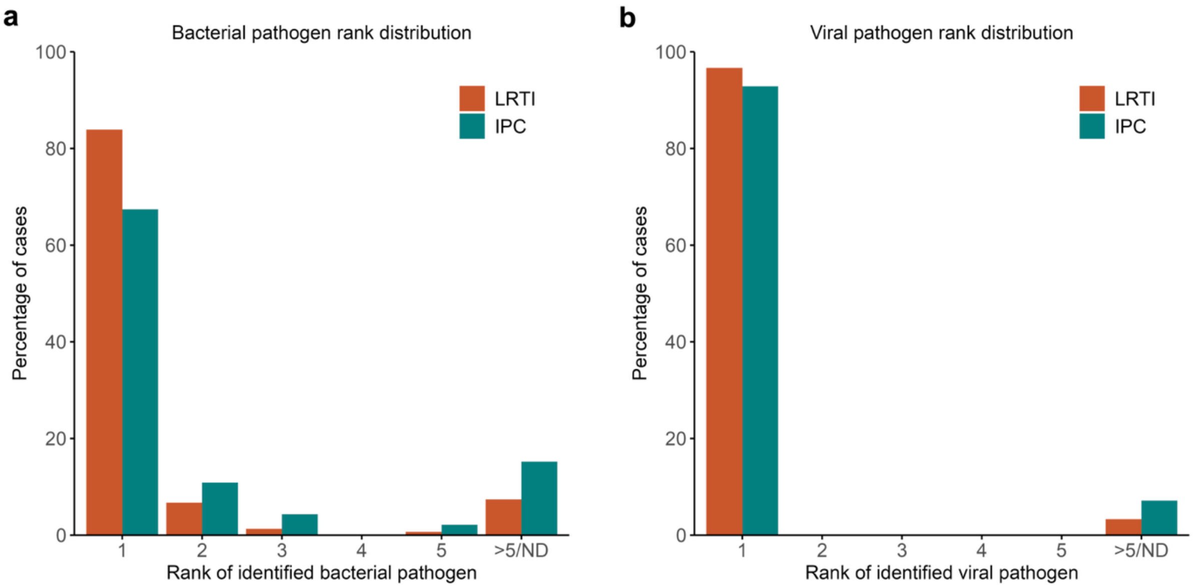

Figure S3. Rank plots of detected bacterial and viral pathogens. a. Bar plot showing the distribution of pathogen ranks in patients who had a bacterial pathogen identified, stratified by LRTI and IPC groups. Rank was determined based on reads per million (RPM) of the detected pathogen in the metatranscriptomics data. If more than one bacterial pathogen was detected in a sample, the rank of the higher ranking pathogen was plotted. Ranks >5 and cases where the implicated pathogen was not identified in the metatranscriptomics data were grouped together as >5/ND. b. Bar plot showing the distribution of pathogen ranks in patients who had a viral pathogen identified, using the same methodology. Bacterial cases included patients with any bacteria detected (bacterial LRTI n=26, bacterial/viral LRTI n=123, bacterial IPC n=28, bacterial/viral IPC n=18; total bacterial n=195), and viral cases included patients with any virus detected (viral LRTI n=58, bacterial/viral LRTI n=123, viral IPC n=24, bacterial/viral IPC n=18; total viral n=223). Figure is descriptive; no statistical tests were performed. Abbreviations: LRTI, lower respiratory tract infection; IPC, incidental pathogen carriage; RPM, reads per million; ND, not detected.

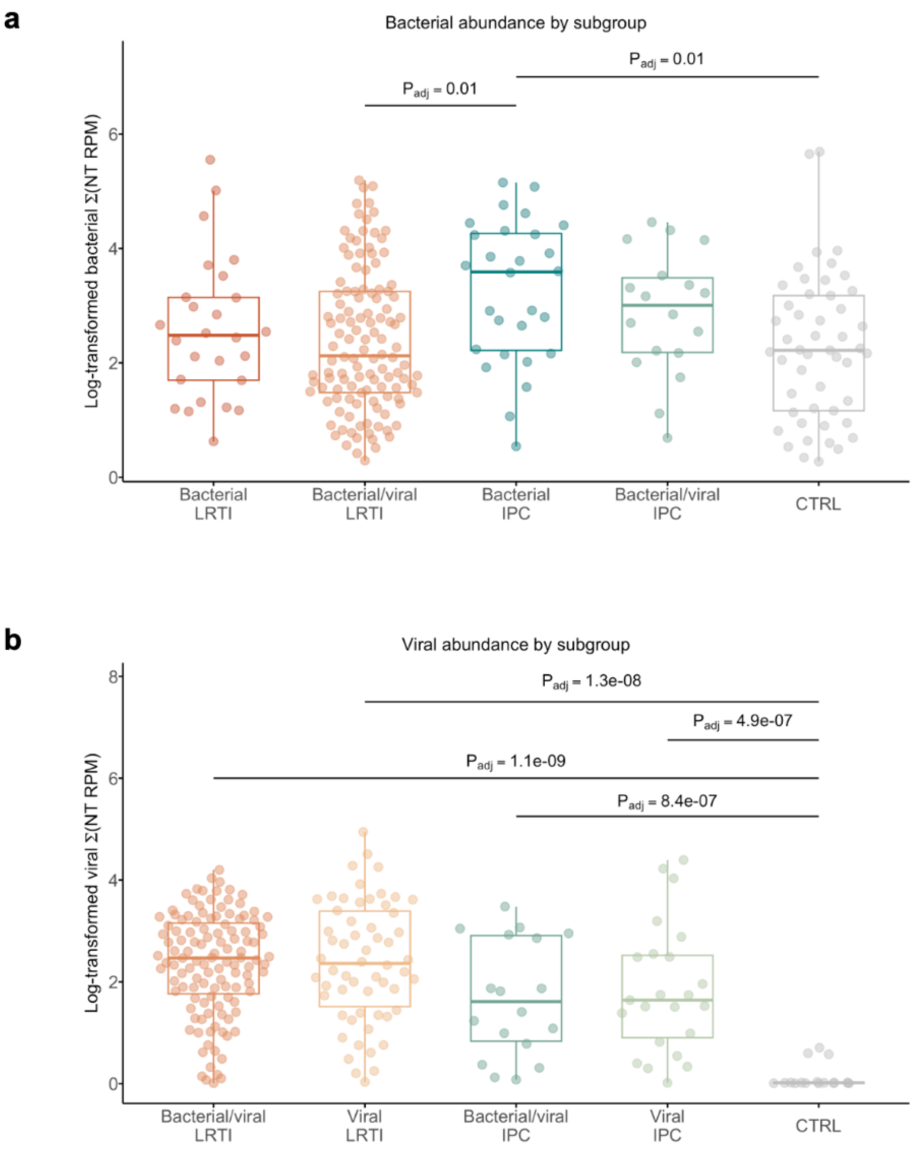

Figure S4. Microbial load by infection subclass. a. Total bacterial abundance measured in reads per million (RPM) across bacterial subclasses and controls (Bacterial LRTI: n=26, Bacterial/viral LRTI: n=120, Bacterial IPC: n=28, Bacterial/viral IPC: n=18, CTRL: n=49). Analysis restricted to patients with bacterial pathogens by composite microbiology and samples with detectable bacterial reads passing background filtering. b. Total viral abundance across viral subclasses and controls (Bacterial/viral LRTI: n=119, Viral LRTI: n=58, Bacterial/viral IPC: n=18, Viral IPC: n=23, CTRL: n=17). Analysis restricted to patients with viral pathogens by composite microbiology and samples with detectable viral reads passing background filtering. P values determined by two-sided Wilcoxon rank-sum tests and adjusted for multiple comparisons using the Benjamin-Hochberg false discovery rate algorithm. Box limits correspond to the IQR with the center line representing the median. The lower whisker extends to the smallest value within (1.5×IQR) below the first quartile, and the upper whisker extends to the largest value within (1.5×IQR) above the third quartile. Abbreviations: LRTI, lower respiratory tract infection; IPC, incidental pathogen carriage, CTRL, control; NT RPM, nucleotide reads per million; IQR, interquartile range.

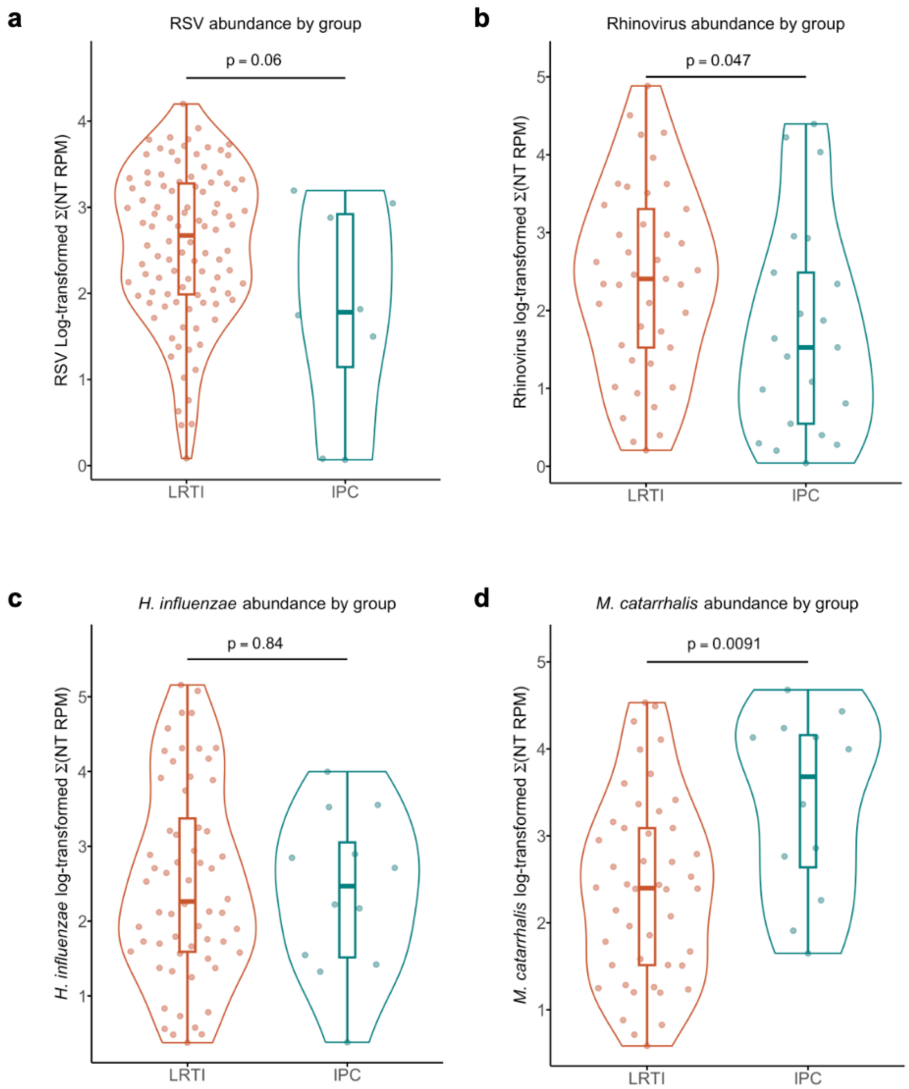

Figure S5. Abundance differences of the most prevalent bacterial and viral pathogens in LRTI versus IPC groups. a. Abundance of RSV, defined as sum of NT RPM, for patients who had RSV detected as a pathogen on mNGS (LRTI: n=100, IPC: n=8). b. Rhinovirus (LRTI: n=41, IPC: n=21). c. *Haemophilus influenzae* (LRTI: n=60, IPC: n=12). d. *Moraxella catarrhalis* (LRTI: n=46, IPC: n=12). P values generated using a two-sided Wilcoxon rank-sum test. For violin plots, the shape of the violin represents the kernel density estimate of the data, with tails trimmed to the upper and lower ranges of the data. Internal box plot box limits correspond to the IQR with the center line representing the median. The lower whisker extends to the smallest value within (1.5×IQR) below the first quartile, and the upper whisker extends to the largest value within (1.5×IQR) above the third quartile. Abbreviations: LRTI, lower respiratory tract infection; IPC, incidental pathogen carriage; RSV, respiratory syncytial virus; IQR, interquartile range; NT RPM, nucleotide reads per million; mNGS, metagenomic next generation sequencing.

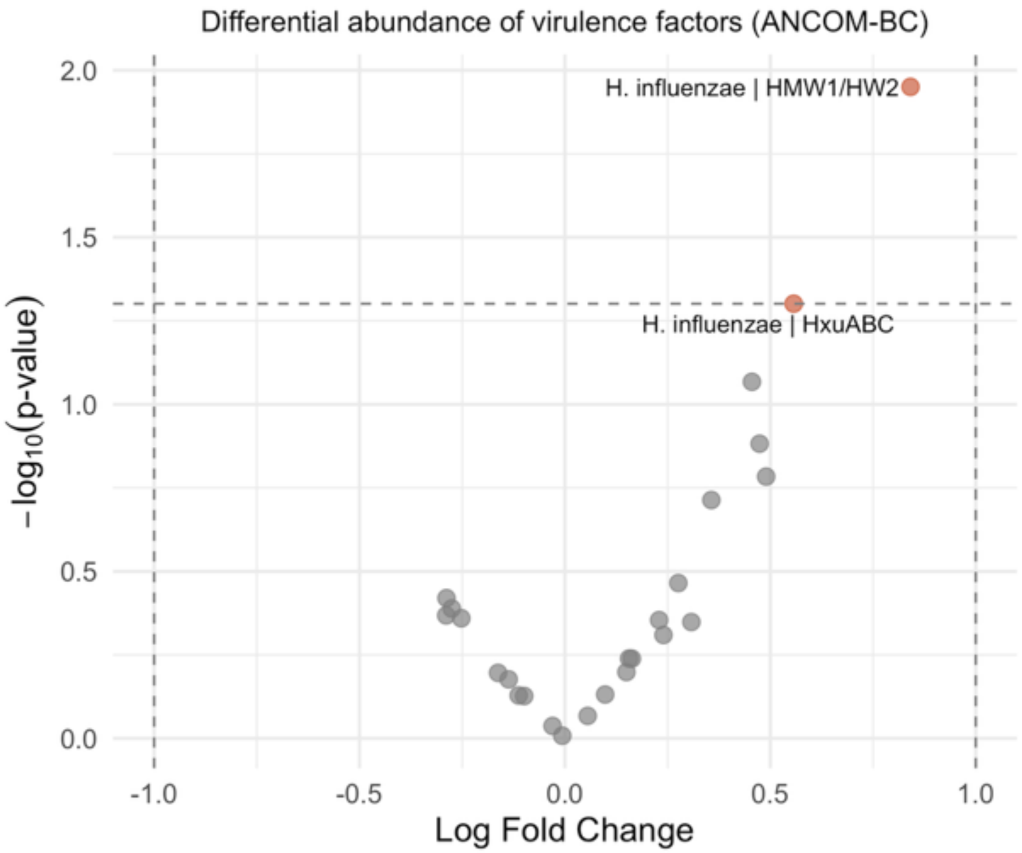

Figure S6. Exploratory analysis of virulence factors in LRTI and IPC groups. Differential abundance of all virulence factors, annotated by VFDB 2.0, between LRTI (n=207) and IPC (n=70) groups, using ANCOM-BC. The fifty most abundant virulence factors by DPM were included. Positive log2 fold change indicates upregulation with LRTI and negative log2 fold change indicates upregulation with IPC. Unadjusted P values generated by ANCOM-BC are shown. Vertical dotted lines correspond to log2 fold change values of -1 and 1. The horizontal dotted line corresponds to the p value cutoff ($p < 0.05$). Colored dots are below the P value threshold, while gray dots are non-significant. Abbreviations: LRTI, lower respiratory tract infection; IPC, incidental pathogen carriage; VFBD, Virulence Factor Database; DPM, depth per million.

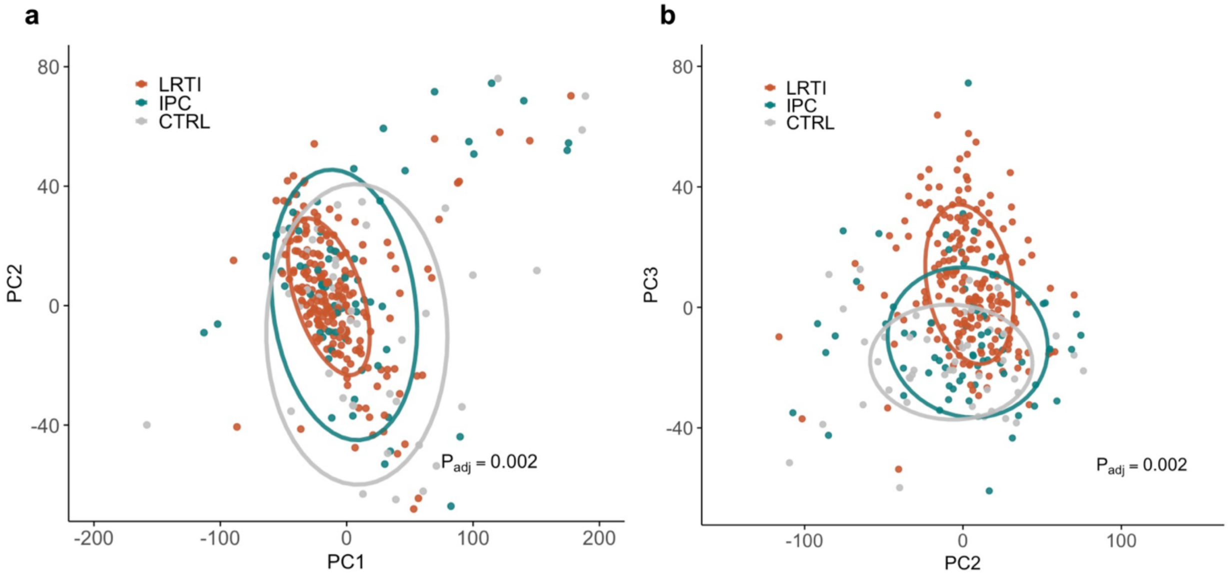

Figure S7. Additional principal components of the lower respiratory tract host transcriptome. PCA of normalized host gene expression profiles from tracheal aspirate samples. a. PC1 versus PC2. b. PC2 versus PC3. Points are colored by clinical group (LRTI, orange; IPC, teal; CTRL, gray). Adjusted P values were calculated using two-sided PERMANOVA comparing LRTI versus non-LRTI groups. All analyses were performed on independent biological samples from individual patients. Sample sizes were LRTI n=207, IPC n=70, and CTRL n=49. Abbreviations: LRTI, lower respiratory tract infection; IPC, incidental pathogen carriage; CTRL, control; PCA, principal component analysis.

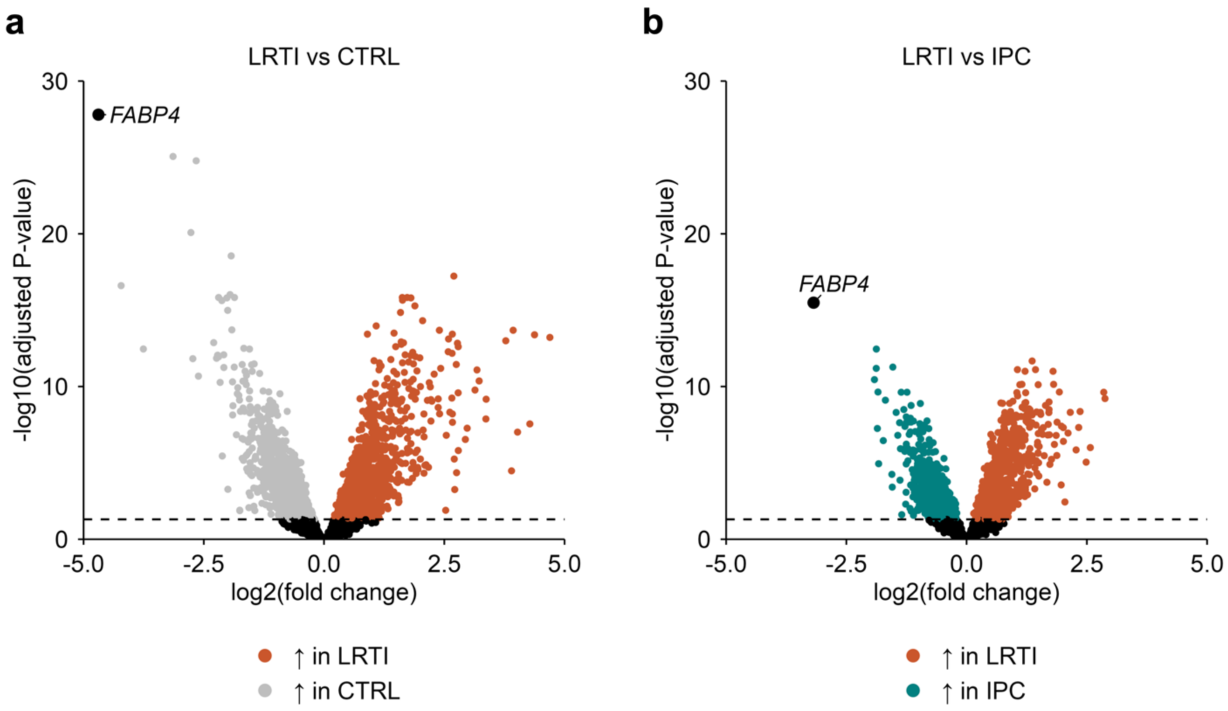

Figure S8. FABP4 is an outlier in differential expression analyses comparing LRTI to CTRL or IPC. Volcano plots showing differential gene expression between a. LRTI and CTRL and b. LRTI and IPC. Each point represents a gene, plotted by $\log_2$(fold change) and $-\log_{10}$(BH-adjusted P value). Differential expression was calculated using limma with moderated two-sided t-tests and Benjamini–Hochberg (BH) multiple-testing correction; genes with $P_{adj}<0.05$ are colored by group (LRTI, orange; CTRL, gray; IPC, teal). FABP4 is labeled and represents an outlier in both comparisons, with among the largest effect sizes and highest statistical significance. All analyses were performed on independent biological samples from individual patients. Sample sizes were LRTI n=207, IPC n=70, and CTRL n=49. Abbreviations: LRTI, lower respiratory tract infection; IPC, incidental pathogen carriage; CTRL, control; BH, Benjamini–Hochberg.

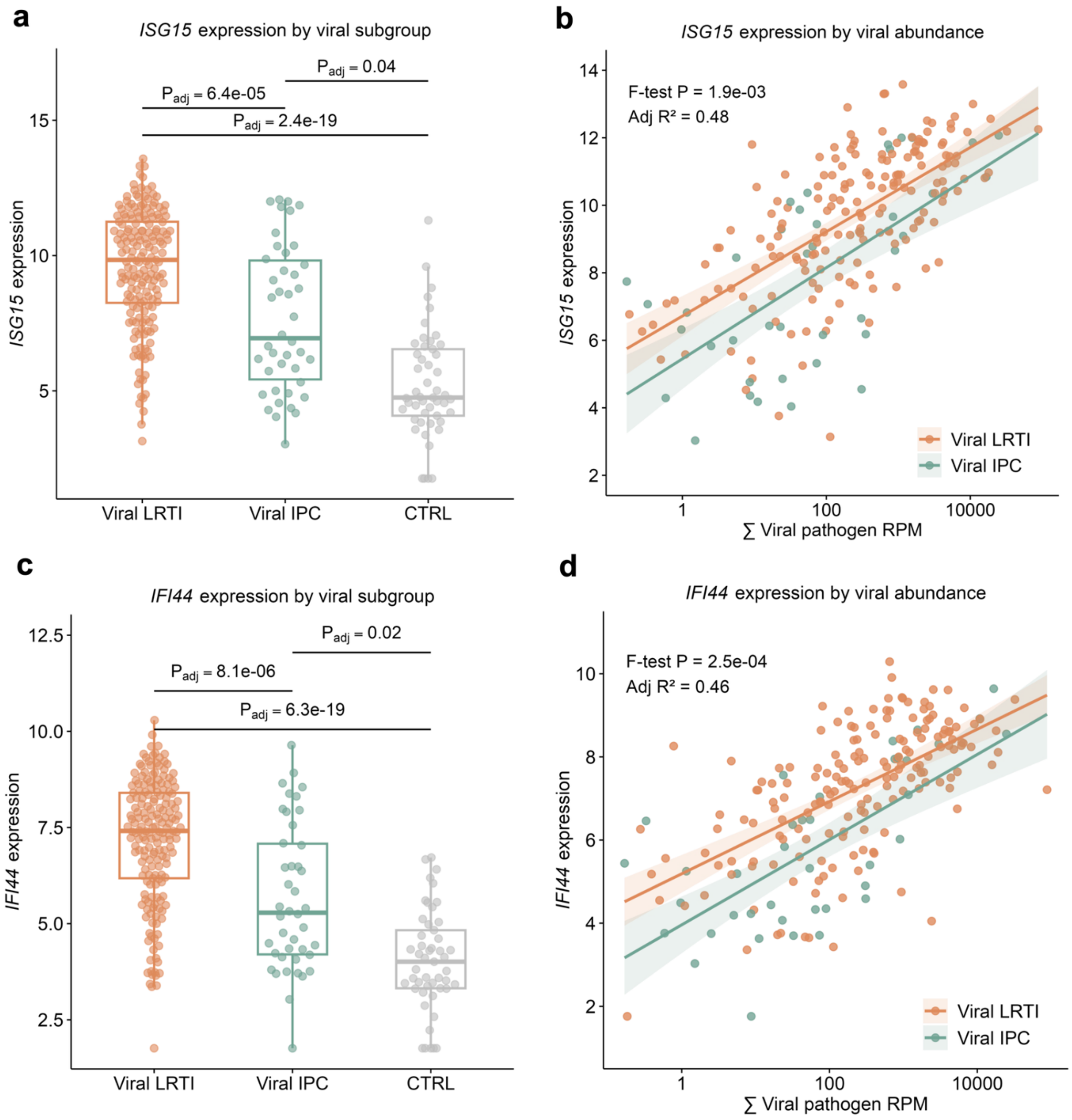

Figure S9. Integrated host–microbial analyses for additional interferon-related genes among viral cases. a. Boxplot of normalized ISG15 expression by group. BH-adjusted P values from differential expression (DE) analyses are shown. b. Linear regression of viral abundance (RPM) against normalized *ISG15* expression for viral LRTI and IPC. c. Boxplot of normalized *IFI44* expression by group. d. Linear regression of viral abundance (RPM) against normalized *IFI44* expression for viral LRTI and IPC. Lines represent least-squares linear regression fits with shaded 95% confidence intervals. DE analyses were performed using limma with moderated two-sided t-tests and BH multiple-testing correction. Box plots show the median (center line), interquartile range (box), and whiskers extending to the most extreme data points within 1.5× the interquartile range. Viral cases included patients with any virus detected by the composite microbiologic definition (viral LRTI n=58, bacterial/viral LRTI n=123, viral IPC n=24, bacterial/viral IPC n=18; total viral n=223). All analyses were performed on independent biological samples from individual patients. Abbreviations: LRTI, lower respiratory tract infection; IPC, incidental pathogen carriage; DE, differential expression; BH, Benjamini–Hochberg; RPM, reads per million; CI, confidence interval.

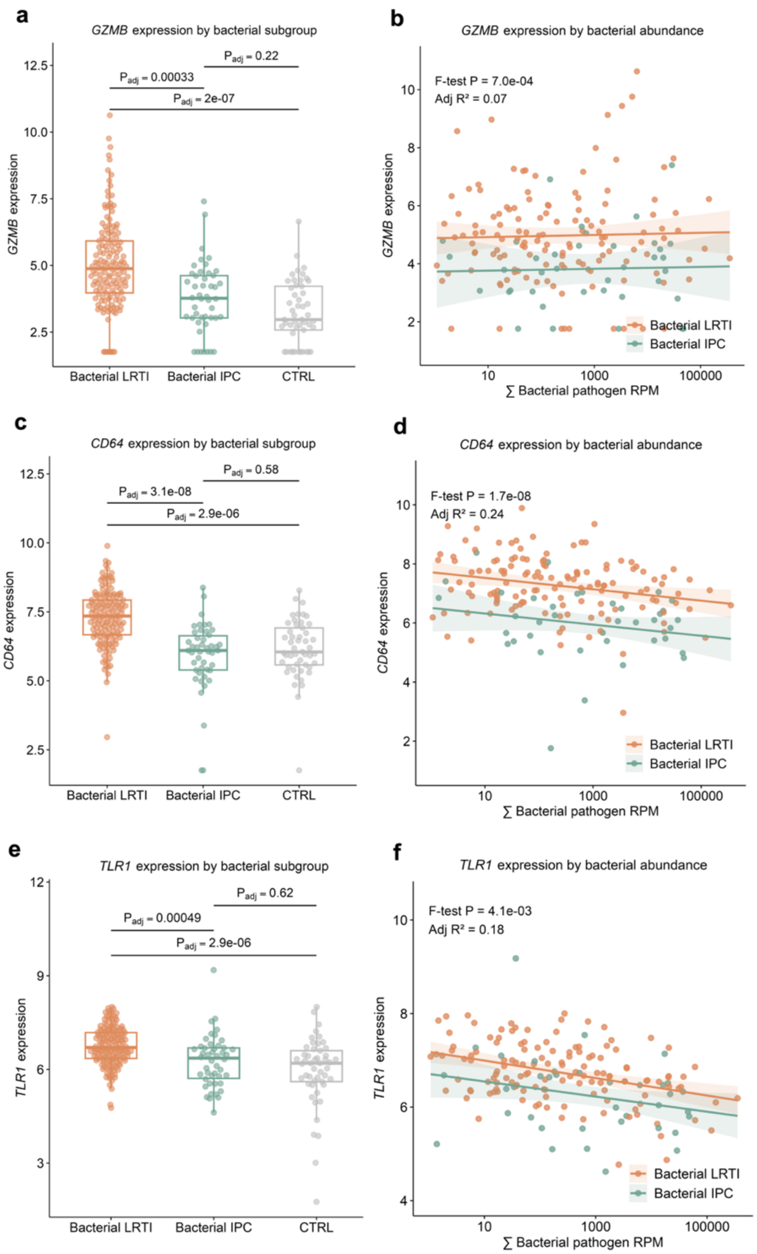

Figure S10. Integrated host–microbial analyses for antibacterial defense genes among bacterial cases. a. Boxplot of normalized *GZMB* expression by group. BH-adjusted P values from DE analyses are shown. b. Linear regression of bacterial pathogen abundance (RPM) against normalized *GZMB* expression for bacterial LRTI and IPC. c. Boxplot of normalized *CD64* expression by group. d. Linear regression of bacterial pathogen abundance (RPM) against normalized *CD64* expression for bacterial LRTI and IPC. e. Boxplot of normalized *TLR1* expression by group. f. Linear regression of bacterial pathogen abundance (RPM) against normalized *TLR1* expression for bacterial LRTI and IPC. DE analyses were performed using limma with moderated two-sided t-tests and BH multiple-testing correction. Box plots show the median (center line), interquartile range (box), and whiskers extending to the most extreme data points within 1.5× the interquartile range. Linear regression lines represent least-squares linear regression fits with shaded 95% confidence intervals. Bacterial cases included patients with any bacteria detected by the composite microbiologic definition (bacterial LRTI n=26, bacterial/viral LRTI n=123, bacterial IPC n=28, bacterial/viral IPC n=18; total bacterial n=195). All analyses were performed on independent biological samples from individual patients. Abbreviations: LRTI, lower respiratory tract infection; IPC, incidental pathogen carriage; DE, differential expression; BH, Benjamini–Hochberg; RPM, reads per million; CI, confidence interval.

2. SUPPLEMENTARY TABLE

Model Feature	ENSEMBL ID	Fold 1	Fold 2	Fold 3	Fold 4	Fold 5
Shannon Diversity Index	N/A	-0.151	-0.241	-0.234	-0.252	-0.231
ZNF12	ENSG00000164631	-0.053	-	-	-	-
WBP2	ENSG00000132471	-	-	-	-	-0.173
VCPIP1	ENSG00000175073	-	-	-	0.229	-
TAP1	ENSG00000168394	-	0.031	-	-	-
SNHG19	ENSG00000260260	-	-	-	-	-0.054
SAP30L	ENSG00000164576	-	-	-	-	0.060
RSAD2	ENSG00000134321	0.099	-	-	-	-
RPS6KA1	ENSG00000117676	-	-	-	-	-0.227
RBP4	ENSG00000138207	-0.225	-0.198	-0.202	-0.085	-0.220
PSMB8	ENSG00000204264	-	-	0.030	-	-
PRKDC	ENSG00000253729	-0.281	-	-0.057	-	-0.207
PPP1R14B	ENSG00000173457	-	-	-	-	-0.151
PIK3CA	ENSG00000121879	0.236	-	-	-	-
PARP9	ENSG00000138496	-	0.059	-	0.122	0.122
NMI	ENSG00000123609	-	-	-	-	0.049
MUC2	ENSG00000198788	0.021	-	-	-	-
MED14	ENSG00000180182	0.063	-	-	-	0.212
MARCO	ENSG00000019169	-0.009	-	-	-	-0.160
IRF7	ENSG00000185507	-	0.198	0.102	0.137	0.166
IRF1	ENSG00000125347	-	0.110	0.118	-	-
IL3RA	ENSG00000185291	0.024	-	-	-	0.075
IFITM1	ENSG00000185885	0.045	-	-	-	-
IFIT2	ENSG00000119922	-	-	0.015	0.009	-
H2BC12	ENSG00000197903	0.031	-	0.003	-	-
H2AC14	ENSG00000276368	0.016	-	-	-	-
GZMB	ENSG00000100453	-	-	0.020	-	-
GNLY	ENSG00000115523	0.121	-	-	-	-
FFAR3	ENSG00000185897	0.285	0.019	0.110	0.131	-
FCGR1A	ENSG00000150337	-	-	-	-	0.050
FABP4	ENSG00000170323	-0.137	-0.094	-0.146	-0.138	-0.070
ERGIC1	ENSG00000113719	-0.217	-	-	-	-
CYBB	ENSG00000165168	-	-	-	-	0.021
CES1	ENSG00000198848	-	-0.010	-	-0.081	-0.108
CDHR3	ENSG00000128536	-	-	-	-	-0.013
CCR1	ENSG00000163823	-	-	-	-	0.040
CASC3	ENSG00000108349	-0.115	-	-	-	-
C5AR2	ENSG00000134830	-	-	-	-	-0.005
C10orf55	ENSG00000222047	-	-	-	-	-0.057
BATF	ENSG00000156127	-	-	-	-	0.144
AP003170.3	ENSG00000256452	-	-	-	-	-0.015
AP000442.1	ENSG00000255139	-	-	-	-	-0.002
AOC3	ENSG00000131471	-0.046	-	-	-0.026	-
AL731567.1	ENSG00000231964	-	-	-	-	0.015
AL627230.1	ENSG00000275493	-	-	-	-	-0.013
ADAMDEC1	ENSG00000134028	0.057	-	-	-	-

Table S1. Integrated multi-gene and Shannon Diversity Index classifier coefficients. Model coefficients for features included in each of five cross-validation folds are shown. Coefficients were derived from LASSO-regularized logistic regression models. Values represent model coefficients (log-odds scale) for each feature within a given fold. Features not selected in a given fold are indicated by a dash. Shannon Diversity Index, FABP4, and RBP4 were selected in all folds.

3. SUPPLEMENTARY METHODS

For this study, patients were classified into three groups:

Group	Retrospective Adjudication	Microbiology
**Lower respiratory tract infection (LRTI)**	Lower respiratory tract infection present	Positive
**Incidental pathogen carriage (IPC)**	Lower respiratory tract infection absent	Positive
**Control (CTRL)**	Lower respiratory tract infection absent	Negative

Retrospective clinical adjudication was performed independently by two or more clinicians with expertise in pediatric infectious diseases and/or intensive care who practice clinically at each enrolling site. Disagreements were resolved by panel review. Adjudicators were instructed to review all clinical, laboratory, microbiology, and radiology data available through the end of the hospital admission, specifically incorporating the clinical variables outlined in the Center for Disease Control (CDC) and National Healthcare Safety Network (NHSN) Pneumonia (PNU) 1 definition into their adjudication:

- Fever (>38.0°C) or hypothermia (<36°C) or temperature instability
- Bradycardia or tachycardia, based on age
- Leukopenia (≤ 4,000 WBC/mm^3)
- Leukocytosis (≥ 12,000 WBC/mm^3 if >12 years old; ≥ 15,000 WBC/mm^3 if <12 years old)
- History of new onset or worsening cough, dyspnea, apnea, or tachypnea
- New onset of purulent sputum, change in character of sputum, increased respiratory secretions, or increased suctioning requirements
- Compatible pulmonary physical exam findings:
    - Rales
    - Rhonchi
    - Wheezing
    - Bronchial breath sounds
    - Nasal flaring
    - Chest wall retractions
    - Grunting
- Worsening gas exchange, based on:
    - O_2 desaturations
    - Low PaO_2:FiO_2 ratio
    - Increased oxygen requirements
    - Increased ventilator demand
- New and persistent or progressive and persistent radiographic findings, including:
    - Infiltrate
    - Consolidation
    - Cavitation
    - Pneumatoceles (for infants ≤ 1 year old)

Microbiology included a combination of standard-of-care clinical microbiologic testing and respiratory metatranscriptomics for comprehensive identification of respiratory pathogens. To be considered positive, clinical microbiology alone, metatranscriptomics alone, or both needed to have pathogens detected.

- Clinical microbiology ordered by treating clinicians from 72 hours before to 48 hours after intubation, including:
    - Multiplex respiratory viral or respiratory pathogen panel from nasopharyngeal swab*
    - Semiquantitative bacterial cultures from sputum, tracheal aspirate, or bronchoalveolar fluid
- Metatranscriptomics performed on tracheal aspirate:
    - Viruses that passed background filtering with abundance > 0.1 RPM with established respiratory pathogenicity
    - Bacteria that passed background filtering and were identified as hits using the established rules-based model (RBM)

*Standard-of-care clinical viral testing was frequently performed on upper respiratory tract specimens, whereas host transcriptomic and microbiome profiling in this study was conducted exclusively on lower respiratory tract samples (tracheal aspirates). Because microbial detection and carriage can differ between upper and lower airway compartments, and because the biological focus of this study was the lower respiratory tract, we specifically reviewed discordance between upper and lower respiratory tract pathogen detection that would have altered microbiologic classification. Such discordance was rare, occurring in only 9 patients (6 LRTI and 3 IPC; 2.9% of LRTI and 4.3% of IPC cases). Notably, in 4 of these 9 cases, the same viral pathogen was detected in lower respiratory tract metatranscriptomic data but fell below predefined abundance and/or background-filtering thresholds. These findings suggest that our microbiologic definitions were robust, reflected lower airway biology, and aligned with how respiratory pathogen detection is interpreted in routine clinical practice.
