# Supplementary material for: Host–microbiome archetypes differentiate infection from pathogen carriage in the human lower airway
Source: Nat Commun. 2026 Apr 13;17:5149. doi: 10.1038/s41467-026-71863-5 (PMC13250161; doi:10.1038/s41467-026-71863-5)
Supplement: Supplementary file 2 — Description of Additional Supplementary Files [file 41467_2026_71863_MOESM2_ESM.pdf]

Description of Additional Supplementary Files

Supplementary Data Files

Supplementary Data 1. ANCOM-BC differential taxonomic abundance between LRTI and IPC.

Supplementary Data 2. Differential virulence factor abundance between LRTI and IPC.

Supplementary Data 3. Metabolic pathway differences between LRTI and IPC.

Supplementary Data 4. Differential gene expression (LRTI vs CTRL; LRTI vs IPC; IPC vs CTRL).

Supplementary Data 5. Gene set enrichment analysis results (LRTI vs IPC; IPC vs CTRL).

Supplementary Data 6. Subgroup differential gene expression analyses (viral and bacterial).

Supplementary Data 7. Differential gene expression adjusted for Shannon diversity index.

Supplementary Data 8. Background contaminants identified in negative controls.
