# Supplementary material for: Host–microbiome archetypes differentiate infection from pathogen carriage in the human lower airway
Source: Nat Commun. 2026 Apr 13;17:5149. doi: 10.1038/s41467-026-71863-5 (PMC13250161; doi:10.1038/s41467-026-71863-5)
Supplement: Supplementary file 11 — Reporting Summary [file 41467_2026_71863_MOESM11_ESM.pdf]

nature portfolio

Corresponding author(s): Charles R. Langelier

Last updated by author(s): 02/04/2026

Reporting Summary

Nature Portfolio wishes to improve the reproducibility of the work that we publish. This form provides structure for consistency and transparency in reporting. For further information on Nature Portfolio policies, see our Editorial Policies and the Editorial Policy Checklist.

Statistics

For all statistical analyses, confirm that the following items are present in the figure legend, table legend, main text, or Methods section.

n/a	Confirmed	
☐	☒	The exact sample size (*n*) for each experimental group/condition, given as a discrete number and unit of measurement
☐	☒	A statement on whether measurements were taken from distinct samples or whether the same sample was measured repeatedly
☐	☒	The statistical test(s) used AND whether they are one- or two-sided<br>*Only common tests should be described solely by name; describe more complex techniques in the Methods section.*
☐	☒	A description of all covariates tested
☐	☒	A description of any assumptions or corrections, such as tests of normality and adjustment for multiple comparisons
☐	☒	A full description of the statistical parameters including central tendency (e.g. means) or other basic estimates (e.g. regression coefficient) AND variation (e.g. standard deviation) or associated estimates of uncertainty (e.g. confidence intervals)
☐	☒	For null hypothesis testing, the test statistic (e.g. *F*, *t*, *r*) with confidence intervals, effect sizes, degrees of freedom and *P* value noted<br>*Give P values as exact values whenever suitable.*
☒	☐	For Bayesian analysis, information on the choice of priors and Markov chain Monte Carlo settings
☒	☐	For hierarchical and complex designs, identification of the appropriate level for tests and full reporting of outcomes
☒	☐	Estimates of effect sizes (e.g. Cohen's *d*, Pearson's *r*), indicating how they were calculated

Our web collection on statistics for biologists contains articles on many of the points above.

Software and code

Policy information about availability of computer code

Data collection	Metatranscriptomic sequencing of tracheal aspirates was performed to profile both host gene expression and airway microbial communities. Microbial reads were processed using the CZ ID platform (Illumina mNGS pipeline v7.1). Host gene expression was quantified from RNA-seq data following pseudoalignment with Kallisto. Protein measurements were obtained using the SomaScan 7k assay (SomaLogic).
Data analysis	All analyses were conducted in R (v4.4.2 and v4.5.0). Microbial abundance comparisons were performed using the rstatix package (v0.7.2). Alpha and beta diversity metrics were calculated using the vegan package (v2.7-1). Differential microbial abundance was assessed using ANCOM-BC (v2.8.1). RNA-seq reads were pseudoaligned using Kallisto, and gene-level counts were generated using tximport (v1.28, Bioconductor 3.18) with the scaled TPM method. Counts were variance-stabilized using DESeq2 (v1.50.2). Differential expression analyses were performed using limma-voom (v3.66.0), and gene set enrichment analysis was conducted using ReactomePA (v1.54.0). Hierarchical clustering and heatmap visualization were performed using ComplexHeatmap (v2.26.1). Mediation analyses were conducted using the mediation package (v4.5.1). Classifier development used glmnet (v4.1-10), and performance was evaluated using pROC (v1.19.0.1). Statistical tests and modeling were performed using base R functions (stats package).

For manuscripts utilizing custom algorithms or software that are central to the research but not yet described in published literature, software must be made available to editors and reviewers. We strongly encourage code deposition in a community repository (e.g. GitHub). See the Nature Portfolio guidelines for submitting code & software for further information.

Data

Policy information about availability of data

All manuscripts must include a data availability statement. This statement should provide the following information, where applicable:

- Accession codes, unique identifiers, or web links for publicly available datasets
- A description of any restrictions on data availability
- For clinical datasets or third party data, please ensure that the statement adheres to our policy

FASTQ files containing the microbial sequencing reads following subtraction of reads aligning to the human genome have been deposited in the NCBI Sequence Read Archive under BioProject accession PRJNA748764 (https://www.ncbi.nlm.nih.gov/bioproject/?term=PRJNA748764). Processed host gene counts, microbial taxon counts, and deidentified clinical metadata are available in the GitHub repository associated with this work: https://github.com/infectiousdisease-langelier-lab/Incidental_pathogen_carriage (https://doi.org/10.5281/zenodo.19078486). Source data for each of the figures is provided in the Supplementary information.

Research involving human participants, their data, or biological material

Policy information about studies with human participants or human data. See also policy information about sex, gender (identity/presentation), and sexual orientation and race, ethnicity and racism.

Reporting on sex and gender	Sex at birth is reported for each study participant.
Reporting on race, ethnicity, or other socially relevant groupings	Self-identified race is reported for each patient, and includes the following groups: Asian, Black, White, Multiple, Other, American Indian/Alaska Native and Native Hawaiian/Pacific Islander. Self-identified hispanic ethnicity is reported for each patient.
Population characteristics	A comprehensive table of population characteristics is provided in Table 1.
Recruitment	Participants were recruited from a prospective multicenter cohort of critically ill, mechanically ventilated children with acute respiratory illness admitted to eight U.S. pediatric intensive care units (CPCCRN) between 2015 and 2017. Eligible patients were identified following intubation and approached consecutively for enrollment if tracheal aspirate samples were obtained within 24 hours. Written informed consent was obtained from legal guardians, with an initial waiver of consent for sample collection. Recruitment was limited to critically ill, intubated children, which may affect generalizability and introduce selection bias; however, consecutive enrollment across multiple sites helped mitigate this.
Ethics oversight	The study was approved by the University of Utah central IRB #00088656.

Note that full information on the approval of the study protocol must also be provided in the manuscript.

Field-specific reporting

Please select the one below that is the best fit for your research. If you are not sure, read the appropriate sections before making your selection.

[x] Life sciences [ ] Behavioural & social sciences [ ] Ecological, evolutionary & environmental sciences

For a reference copy of the document with all sections, see nature.com/documents/nr-reporting-summary-flat.pdf

Life sciences study design

All studies must disclose on these points even when the disclosure is negative.

Sample size	No sample size calculation was preformed as we leveraged all available data from a large (N=457) multicenter observational cohort. Many microbiome and transcriptomic studies have a much smaller population size, and our sample size was robust enough to identify several significant findings.
Data exclusions	Data from every patient eligible for the study was analyzed.
Replication	Each participant contributed a single biological sample, and no technical replicates were performed. Findings were derived from this single cohort, and no independent external dataset was available for replication.
Randomization	This was an observational study and thus no randomization was performed.
Blinding	Clinical adjudication of infection status was performed retrospectively by two independent physician reviewers who were blinded to metatranscriptomic sequencing results, with disagreements resolved by consensus review.

Reporting for specific materials, systems and methods

We require information from authors about some types of materials, experimental systems and methods used in many studies. Here, indicate whether each material, system or method listed is relevant to your study. If you are not sure if a list item applies to your research, read the appropriate section before selecting a response.

Materials & experimental systems

n/a	Involved in the study
☒	☐ Antibodies
☒	☐ Eukaryotic cell lines
☒	☐ Palaeontology and archaeology
☒	☐ Animals and other organisms
☒	☐ Clinical data
☒	☐ Dual use research of concern
☒	☐ Plants

Methods

n/a	Involved in the study
☒	☐ ChIP-seq
☒	☐ Flow cytometry
☒	☐ MRI-based neuroimaging

Plants

Seed stocks	N/A
Novel plant genotypes	N/A
Authentication	N/A
