# Supplementary material for: Host–microbiome archetypes differentiate infection from pathogen carriage in the human lower airway
Source: Nat Commun. 2026 Apr 13;17:5149. doi: 10.1038/s41467-026-71863-5 (PMC13250161; doi:10.1038/s41467-026-71863-5)
Supplement: Supplementary file 12 — Transparent Peer Review file [file 41467_2026_71863_MOESM12_ESM.pdf]

nature portfolio

# Peer Review File

# Host–Microbiome Archetypes Differentiate Infection from Pathogen Carriage in the Human Lower Airway

Corresponding Author: Dr Charles Langelier

**This file contains all reviewer reports in order by version, followed by all author rebuttals in order by version.**

Version 0:

Reviewer comments:

Reviewer #1

(Remarks to the Author)
This manuscript comes from one of the World's leading groups in this specific field of infectious diseases. Its authors asked whether a combined metagenomic investigation of both host and pathogen in 326 ventilated children could offer mechanistic and diagnostic insights into whether their respiratory failure was considered to represent pneumonia driven by pathogenic organisms (LRTI), or merely accompanied by them (IPC). A third control group consisted of children whose respiratory failure was associated with explainable pathologies other than infection and no evidence of pathogen (CTRL).
These children were indexed into these three groups by clinical assessors blinded to these metagenomic results.
They demonstrate that LRTI is associated with both reduced taxonomic richness and lower transcriptional signatures of innate and adaptive response, whilst IPC was associated with greater bacterial abundance, to include anaerobes as well as higher transcriptional innate immune and adaptive responses, the latter similar to controls. They also went on to demonstrate significant associations between elements of the airway microbiome and the host response to pathogens. Finally, they demonstrated that a combination of host and pathogen metagenomic analysis led to impressive discrimination between LRTI and both IPC and control patients, and that measurement of a single macrophage gene product, FABP4, was equally capable of differentiating between "true" lung infection and that caused by other factors, whether or not there was evidence of "bystander" pathogens.
This is truly a Magnum opus, and could have been split into possibly 3 separate publications. The dataset these authors gathered and display is truly gargantuan and catholic in its origins and breadth. I suspect that the authors wished to keep the considerably different research elements within this study together, to build a more coherent story of the very complicated landscape of both the microbiome and how local immune responses relate to it, both when pathogens are present and not.
It is a difficult paper to read and this referee took three attempts at it to fully digest its contents as it is so data-dense. It is thankfully very well written.
It also represents in my opinion a very significant development in our understanding of the pathology of infectious diseases, both bacterial and viral, as they come to be associated with (or cause) respiratory failure.
This is an exceptional paper, all the more so because it addresses a real "block" in our understanding of pulmonary infectious pathology.
The "block" relates to the unresolved question of whether patients may be "dying of" a pulmonary pathogen, or "dying with" a relative bystander, not responsible for the underlying respiratory failure. This is a perennial question, asked almost daily in ICUs worldwide. Because we don't know (as an example) what a heavy growth of Klebsiella spp. returned from a ventilated patient's lungs means that we treat with antibiotics "just in case". I note that 99% of patients enrolled in this study were given empiric antibiotics – despite little if any evidence that Koch's postulates have ever been fulfilled for this and many related organisms, almost all them Gram negative, in the setting of ICU ventilation. Such is the current Gestalt of this clinical scenario. Such prescriptions are typical, traditional "ICU culture" in such a setting and are doubtless responsible for a very significant contribution to antimicrobial resistance. The issue is that such prescriptions may not be necessary in the first place for at least some patients. The authors address this specifically in the particularly interesting group they describe as "incidental pathogen carriage" – a group which will almost by definition receive empiric antibiotics as whether or not the pathogens isolated are causing trouble is not known.

This paper begins to shed light on a more evidence-based approach to this very big problem, with clear implications for both clinical practice and the advancement of knowledge in this area.

This study demonstrates that a decision boundary between "true pathogens" and the same organisms being described as

“colonisers” can be objectively constructed.
It furthermore highlights the loss of diversity associated with true infection, as well as its relationship to innate and adaptive immune response.

There are also other data relating to both the association between H influenzae virulence factors and true LRTI, as well as differences in the metabolomics of their three clinically indexed groups.

Some data relating to a “metabolic analysis”, offers interesting and additional insight into the condition of “incidental pathogen carriage”.

The discussion is (for the huge amount of data presented) in my opinion very well “distilled” and not only very readable, but appropriate in that it always retains a connection to the clinical challenges posed by this condition, often detecting pathogens whose role in the process is “inferred” but actually unknown and with a remarkably poor level of mechanistic insight and understanding.

I cannot comment on the appropriateness, correct deployment and interpretation of some of the statistical methods, however.

I have no major criticisms of this manuscript from my perspective as an academic clinician. But I confirm that with this study, the authors have addressed one of the most important and unresolved issues in the field of crtical pulmonary illness and infection - namely, the relasiosphips between these two and the association with the ever increasing evidence of the importance of the microbiome in such processes.

Perhaps the biggest area for uncertainty relates to the clinical indexing of the cases – as whether or not the detection of a particular pathogen implies that it is driving, as opposed to accompanying, disease remains currently a decision down to clinical interpretation of the case in the round. In that sense, I suspect that this represents the weakest part of the study – but paradoxically it is because this is a difficult area, that this work was performed in the first place. Always difficult to tighten up a study with a better “gold standard” which is why the work is being done in the first place.

As concerns other matters, these are all minor and more suggestions:

1. Perhaps the authors might consider describing their interpretation of the bimodal distributions in Figure 2 (g)? Might this somehow relate to antibiotic effect? Or have I misinterpreted the graph? Either way, this might need a bit more explanation.
2. This study was in children – the authors correctly point out that adults with the same conditions might behave differently – and I think the authors are right to point this out very clearly.
3. They ask the fascinating question as to whether diversity collapse before, during or after infection. Some very early (maybe about 30 years ago) ICU UK studies with conventional microbiology demonstrated that the appearance of “abnormal” bacterial dominance in the airways preceded clinical deterioration by a few days. This was a tantalising finding at the tie – and I wonder whether this might be referenced? This was not my work, by the way and this is a suggestion only.
4. Equally, a very early study of trucut lung biopsies in people who had just died of VAP were cultured conventionally, alongside histology and the last Chest X ray available. This paper was considered counterintuitive at the time and demonstrated no relationship between pulmonary shadowing, organism density and histological evidence of infection at specific lung sites. There may be some elements of these findings that may be of interest to consider here. I appreciate that these are old data and in adults, and I am simply mentioning these for the authors to consider. The paper is at: DOI: 10.1097/00000542-199604000-00002. I note that at the time, the paper was largely "ignored" - as its findings made those who belived that Kich's postulates for any bacterium finding itself in the lung were satisfied and that antibiotics were necessary. Such a paradigm sadly continues to this day.
5. Finally, the authors highlight the appearance of anaerobes in their IPC cohort. Some intensivists “believe” in the role of these organisms as true lung pathogens in the absence of evidence of aspiration and others don’t. They might perhaps consider discussing this a bit further – specially since anaerobic LPS if anything antagonises macrophage activation when compared to “ordinary” LPS.

Other than these minor points, the authors are to be congratulated for the very considerable clinical and academic relevance of this work. I look forward to the same approach being applied to an adult population as I struggle to consider whether results will be significantly different, or not. Either way, I suspect that this this will also be a very important publication.

Finally, the work prompts the possibility of macrophage FABP4 as a differential diagnostic in thsi clinical space. Shooudl this prove clicnially successful, thsi will be a very major advance in the limitation of inapprorpiate antibitois in this setting; such a finding would be an excellent example of successful translation from CLinical Science to Practice.

(Remarks on code availability)

Reviewer #2

(Remarks to the Author)
In this study, Lydon et al., evaluated the metatranscriptome of tracheal aspirate from 326 mechanically ventilated children with clinically adjudicated LRTI (n=207), IPC (n=70), and non-infectious acute respiratory illnesses (n=49).
There are several strengths here. This study tries to address an important problem, where incidental pathogen carriage frequently happens and we do not know how to interpret or what to do. This culture independent method seems to provide a new view on this issue. The use of metatranscriptomic RNA sequencing is a cutting edge unfrequently used technique for lower respiratory tract studies.
Below are my point-by-point comments.

Major:

1. In abstract: “The single gene FABP4, when combined with alpha diversity, performed similarly, and FABP4 protein alone achieved an AUC=0.88 (95% CI 0.82–0.93).”. A mention of what FABP4 would facilitate the reading of the abstract
2. A key factor here is the presence or absence of clinical LRTI as described in the methods. One can probably infer what that means but there might be some room for difference of criteria. For example, was this only based on imaging, or changes in oxygeneation patter, fever, leukocytosis, etc., were also considered. They should add this criteria in the methods.
3. In some patients nasopharyngeal swabs were utilized for identification of respiratory pathogen. Those could have nasal incidental pathogen carriage and not lower respiratory tract incidental pathogen carriage. Those are two distinct scenarios. Did the authors try to separate the two?
4. Line 118: “Antibiotic usage prior to intubation was similar across…” A different an important data is to evaluate antibiotic usage prior to sampling as this could modify the evaluation of the metatranscriptome. This could then be used as a confounder to adjust some analyses.
5. Figure 1 shows “Distribution of pathogens among patients with clinically adjudicated lower respiratory tract infection (LRTI) or incidental pathogen carriage (IPC). Bar plot demonstrating the proportion of participants (…) with each detected pathogen as determined by combined clinical testing and metatranscriptomics”. This is bit problematic since there is likely variability of the clinical testing performed across different patients (for example, not every patient might have gotten a respiratory viral panel, or anaerobic cultures, etc.). While this is un avoidable, I would recommend to report the results of clinical testing separately from metatranscriptome.
6. There is no data shown on background (technical) control samples which is commonly needed in low biomass studies.
7. Line 147: “Community composition also differed between LRTI, IPC, and CTRL groups based on the Bray-Curtis index (P<0.001) (Figure 2c).” In the supplement in fig S6 they also report a beta diversity plot comparing these three groups but the distribution is very different. Please clarify.
8. Among the control patients, some might have had a pathogen identified as such based on their criteria on metatranscriptome data. How many patients had that and how does that compare with IPC and LRTI?
9. Line 148: “Despite these distinct microbiome archetypes, the detected pathogen was typically the most abundant microbe in the airway microbiome in both LRTI and IPC (Figure S2)”. Is the detection of pathogen here based on clinical results or based on metatranscriptome relative abundance. If the later were included then these results are kind of obvious, no?
10. For Fig 3b I would expect to see some sort of dendogram supporting the clustering.
11. Line 208: “Among the genes DE between LRTI and both CTRL and IPC groups, we noted that FABP4, which is expressed in macrophages and encodes a lipid chaperone that modulates leukotriene stability38, was a clear outlier in both fold change and statistical significance (Figure 3f).” What criteria supports that FABP4 is a clear outlier (or extreme value). This could be shown by indicating where this gene fall within the volcano plot
12. Line 337: “Unexpectedly, total bacterial abundance was greater in IPC than in LRTI or controls, which may be explained by the enrichment of commensal taxa such as Prevotella, Neisseria, and Porphyromonas and reflect a more resilient and balanced barrier microbiota.” A quantitative method such as ddPCR with a clear target (e.g., 16S rRNA gene) would be more desirable to address this issue.

Minor:
13. In Abstract: “Together, our findings reveal distinct ecological and immunologic archetypes that define LRTI and IPC, and support data-driven, biology-informed LRTI diagnostics that incorporate host and microbe.” Maybe “that incorporate host and microbe signatures” or “biomarkers”
14. Table 1 should be referenced in the text, probably in the third paragraph of the results.

(Remarks on code availability)
seems appropriate thought I did not try to run it

Version 1:

Reviewer comments:

Reviewer #1

(Remarks to the Author)
I thank the authors for their responses to my questions and suggestions. I am happy with their responses and their altered MS text and additions. This MS is in my opinion ready for publication, at least from my standpoint as a clinician rather than a statistician.

(Remarks on code availability)

Reviewer #2

(Remarks to the Author)
The authors have addressed my comments.

(Remarks on code availability)
I briefly reviewed it. It looks pretty clean but I do not have the time to run in on my side.

**Open Access** This Peer Review File is licensed under a Creative Commons Attribution 4.0 International License, which permits use, sharing, adaptation, distribution and reproduction in any medium or format, as long as you give appropriate credit to the original author(s) and the source, provide a link to the Creative Commons license, and indicate if changes were made.
In cases where reviewers are anonymous, credit should be given to 'Anonymous Referee' and the source.
The images or other third party material in this Peer Review File are included in the article's Creative Commons license, unless indicated otherwise in a credit line to the material. If material is not included in the article's Creative Commons license and your intended use is not permitted by statutory regulation or exceeds the permitted use, you will need to obtain permission directly from the copyright holder.
To view a copy of this license, visit https://creativecommons.org/licenses/by/4.0/

**Response to Reviewers' Comments**

**Reviewer #1:**

C1. This manuscript comes from one of the World's leading groups in this specific field of infectious diseases. Its authors asked whether a combined metagenomic investigation of both host and pathogen in 326 ventilated children could offer mechanistic and diagnostic insights into whether their respiratory failure was considered to represent pneumonia driven by pathogenic organisms (LRTI), or merely accompanied by them (IPC). A third control group consisted of children whose respiratory failure was associated with explainable pathologies other than infection and no evidence of pathogen (CTRL).

These children were indexed into these three groups by clinical assessors blinded to these metagenomic results.

They demonstrate that LRTI is associated with both reduced taxonomic richness and lower transcriptional signatures of innate and adaptive response, whilst IPC was associated with greater bacterial abundance, to include anaerobes as well as higher transcriptional innate immune and adaptive responses, the latter similar to controls. They also went on to demonstrate significant associations between elements of the airway microbiome and the host response to pathogens. Finally, they demonstrated that a combination of host and pathogen metagenomic analysis led to impressive discrimination between LRTI and both IPC and control patients, and that measurement of a single macrophage gene product, FABP4, was equally capable of differentiating between "true" lung infection and that caused by other factors, whether or not there was evidence of "bystander" pathogens.

This is truly a Magnum opus, and could have been split into possibly 3 separate publications. The dataset these authors gathered and display is truly gargantuan and catholic in its origins and breadth. I suspect that the authors wished to keep the considerably different research elements within this study together, to build a more coherent story of the very complicated landscape of both the microbiome and how local immune responses relate to it, both when pathogens are present and not.

It is a difficult paper to read and this referee took three attempts at it to fully digest its contents as it is so data-dense. It is thankfully very well written.

It also represents in my opinion a very significant development in our understanding of the pathology of infectious diseases, both bacterial and viral, as they come to be associated with (or cause) respiratory failure.

This is an exceptional paper, all the more so because it addresses a real "block" in our understanding of pulmonary infectious pathology.

The "block" relates to the unresolved question of whether patients may be "dying of" a pulmonary pathogen, or "dying with" a relative bystander, not responsible for the underlying respiratory failure. This is a perennial question, asked almost daily in ICUs worldwide. Because we don't know (as an example) what a heavy growth of Klebsiella spp. returned from a ventilated patient's lungs means that we treat with antibiotics "just in case". I note that 99% of patients enrolled in this study were given empiric antibiotics – despite little if any evidence that Koch's postulates have ever been fulfilled for this and many related organisms, almost all them Gram negative, in the setting of ICU ventilation. Such is the current Gestalt of this clinical

scenario. Such prescriptions are typical, traditional “ICU culture” in such a setting and are doubtless responsible for a very significant contribution to antimicrobial resistance. The issue is that such prescriptions may not be necessary in the first place for at least some patients. The authors address this specifically in the particularly interesting group they describe as “incidental pathogen carriage” – a group which will almost by definition receive empiric antibiotics as whether or not the pathogens isolated are causing trouble is not known.

This paper begins to shed light on a more evidence-based approach to this very big problem, with clear implications for both clinical practice and the advancement of knowledge in this area.

This study demonstrates that a decision boundary between “true pathogens” and the same organisms being described as “colonisers” can be objectively constructed.
It furthermore highlights the loss of diversity associated with true infection, as well as its relationship to innate and adaptive immune response.

There are also other data relating to both the association between H influenzae virulence factors and true LRTI, as well as differences in the metabolomics of their three clinically indexed groups.

Some data relating to a “metabolic analysis”, offers interesting and additional insight into the condition of “incidental pathogen carriage”.

The discussion is (for the huge amount of data presented) in my opinion very well “distilled” and not only very readable, but appropriate in that it always retains a connection to the clinical challenges posed by this condition, often detecting pathogens whose role in the process is “inferred” but actually unknown and with a remarkably poor level of mechanistic insight and understanding.

I cannot comment on the appropriateness, correct deployment and interpretation of some of the statistical methods, however.

I have no major criticisms of this manuscript from my perspective as an academic clinician. But I confirm that with this study, the authors have addressed one of the most important and unresolved issues in the field of crtical pulmonary illness and infection - namely, the relasioships between these two and the association with the ever increasing evidence of the importance of the microbiome in such processes.

Response. We thank Reviewer 1 for their thoughtful and detailed evaluation of our manuscript.

As the reviewer aptly notes, distinguishing whether critically ill patients are “dying of” a pulmonary pathogen or “dying with” a detected bystander organism is a ubiquitous yet unresolved problem in the ICU, with major implications for clinical decision-making and antimicrobial stewardship. Despite its frequency in practice, this question has remained relatively understudied using integrated, biology-informed approaches.

We appreciate that our objective of moving beyond pathogen detection alone and toward an objective framework for differentiating true LRTI from incidental pathogen carriage resonated with the reviewer. We agree that this is a multifaceted problem and intentionally retained complementary analyses within a single manuscript to present an integrated view of host–microbe interactions in this clinical context.

C2. Perhaps the biggest area for uncertainty relates to the clinical indexing of the cases – as whether or not the detection of a particular pathogen implies that it is driving, as opposed to accompanying, disease remains currently a decision down to clinical interpretation of the case in the round. In that sense, I suspect that this represents the weakest part of the study – but paradoxically it is because this is a difficult area, that this work was performed in the first place. Always difficult to tighten up a study with a better “gold standard” which is why the work is being done in the first place.

Response. We agree with the reviewer that clinical adjudication of LRTI versus non-infectious respiratory failure is inherently imperfect and that no true gold standard exists for diagnosing pneumonia in critically ill patients. In the absence of an objective reference standard, we employed a rigorous retrospective clinical adjudication process involving independent review by ≥2 physicians with expertise in pediatric critical care and infectious diseases, performed after ICU discharge, with an additional adjudicator added if needed to obtain consensus on any disagreements.

To further enhance transparency and to also address a similar point raised by Reviewer 2, we have added a new **Supplementary Methods** section detailing the clinical features considered during adjudication, which align with the CDC/NHSN PNEU 1 guidelines that we have cited in our methods and results sections. We have also added a statement to the discussion explicitly acknowledging the limitations of clinical adjudication alongside with a citation that critically examines the use of adjudicated clinical syndromes in diagnostic studies (PMID 36262037, now reference #64):

> Line 417: “First, LRTI status was defined using retrospective clinical adjudication performed following ICU discharge, an approach taken due to the absence of a true gold standard for pneumonia diagnosis[64]. Although adjudication was performed rigorously by ≥2 independent expert reviewers and consensus resolution, some misclassification is unavoidable and reflects the clinical uncertainty this study seeks to address”.

We agree with the reviewer that this uncertainty represents a both a limitation of our study and a central reason why data-driven, biology-informed approaches are needed to distinguish true infection from incidental pathogen carriage.

C3. Perhaps the authors might consider describing their interpretation of the bimodal distributions in Figure 2 (g)? Might this somehow relate to antibiotic effect? Or have I misinterpreted the graph? Either way, this might need a bit more explanation.

Response. We appreciate the reviewer drawing attention to this point and agree that additional clarification is helpful. Figure 2g is a raincloud plot that depicts pathogen burden, defined as the proportion of non-human sequencing reads assigned to the implicated respiratory pathogen(s). A value near 1.0 indicates that the pathogen makes up nearly the entire microbial community (i.e. ecological dominance), whereas lower values near 0.0 indicate that the pathogen represented only a minority of the community among a background microbiome.

Although both LRTI and IPC spanned the full range of pathogen burden/dominance, the distributions differed. LRTI more frequently demonstrated pathogen-dominant communities, and IPC more often reflected scenarios in which the pathogen was just one member of a broader microbial ecosystem. We interpret this to mean even when the same pathogen is detected, the ecological context in which it exists differs between groups, with LRTI characterized by microbiome collapse and loss of diversity, while IPC characterized by a more intact and

balanced microbial community. We view this dominance metric as providing complementary but distinct information from our other microbiome analyses, including abundance (Fig 2d-e, Fig S4), alpha diversity (Fig 2a), and pathogen rank within the community (Fig S3).

The reviewer queried whether preceding antibiotic administration could explain this pattern. Similar proportions of patients received antibiotics prior to intubation in each group (34.8% in LRTI vs 24.3% in IPC, p=0.14, as noted in Table 1), so we suspect that antibiotics alone do not explain the observed pattern. However, preceding antibiotics could contribute to variability seen in each group.

To improve clarity and interpretation, we have revised and expanded upon the following proportion of our results section:

> Line 173: "We subsequently evaluated pathogen burden, quantified as the proportion of non-human sequencing reads assigned to the implicated respiratory pathogen. A value near 1.0 indicates that the pathogen comprised nearly all microbial reads, while lower values (e.g. <0.2) indicate that the pathogen represented only a minority of the community. Although both groups demonstrated a wide range of pathogen burden, patients with LRTI were more likely to exhibit more pathogen-dominant communities, whereas in IPC, the pathogen more often comprised only a small fraction of the microbial community (63.4% versus 36.6% mean pathogen proportion, P=5.9e-7) (**Figure 2g**)."

C4: This study was in children – the authors correctly point out that adults with the same conditions might behave differently – and I think the authors are right to point this out very clearly.

Response. We thank the reviewer for this comment and agree. As we note in the discussion, because we focused exclusively on children (a population with high baseline prevalence of IPC), it remains known whether our findings generalize to adults. We have now also emphasized this as a necessary future direction and are planning parallel analyses in adult cohorts.

> Line 440: "Because this work was conducted in a pediatric cohort, future studies in adult populations will be important to determine whether the microbial and host response patterns that define LRTI and IPC are conserved across the age spectrum."

C5. They ask the fascinating question as to whether diversity collapse before, during or after infection. Some very early (maybe about 30 years ago) ICU UK studies with conventional microbiology demonstrated that the appearance of "abnormal" bacterial dominance in the airways preceded clinical deterioration by a few days. This was a tantalising finding at the tie – and I wonder whether this might be referenced? This was not my work, by the way and this is a suggestion only.

Response. We appreciate the reviewer bringing this important work to our attention. The early ICU study referenced (A'Court et al., 1993, PMID 8255961) used conventional culture methods and found abnormal bacterial dominance in the airways leading up to clinical pneumonia diagnosis, a result that resonates with our observation that pathogen dominance differentiates LRTI from incidental bacterial carriage. Although the methods differ from our sequencing-based approach, the conceptual parallel is striking and adds important historical context to our findings.

Furthermore, this question has also been explored using contemporary molecular profiling. In a prior study from our group examining ventilator-associated pneumonia (VAP), longitudinal respiratory sampling demonstrated that early shifts in community composition and reduced diversity preceded clinical VAP diagnosis (Mourani et al., 2021, PMID 33008935). This study was already cited in the Introduction in the context of critical illness-associated microbiome disruption; in response to the reviewer's comment, we now also reference it in the Discussion to highlight its relevance to the temporal relationship between microbiome changes and infection onset.

Together, these studies support the biological plausibility that microbiome disruption may occur prior to overt infection, while underscoring the need for dedicated longitudinal designs to more precisely define the timing and causality of these changes. We have now referenced both studies in our discussion section, as suggested by the reviewer:

> Line 429: "Longitudinal sampling could help determine whether diversity collapse precedes, accompanies, or follows infection onset, a question that has been raised by early ICU studies using conventional microbiology that suggested abnormal bacterial dominance may precede clinical LRTI diagnosis by several days[65], and supported by more recent sequencing-based studies demonstrating decreased community diversity prior to ventilator-associated pneumonia diagnosis[25]."

C6. Equally, a very early study of trucut lung biopsies in people who had just died of VAP were cultured conventionally, alongside histology and the last Chest X ray available. This paper was considered counterintuitive at the time and demonstrated no relationship between pulmonary shadowing, organism density and histological evidence of infection at specific lung sites. There may be some elements of these findings that may be of interest to consider here. I appreciate that these are old data and in adults, and I am simply mentioning these for the authors to consider. The paper is at: DOI: 10.1097/00000542-199604000-00002. I note that at the time, the paper was largely "ignored" - as its findings made those who belived that Kich's postulates for any bacterium finding itself in the lung were satisfied and that antibiotics were necessary. Such a paradigm sadly continues to this day.

Response: We thank the reviewer for calling this important and underappreciated study to our attention. In that study (Fàbregas et al., 1996, PMID 8638829), conventional lung cultures demonstrated poor correlation with histologically confirmed pneumonia, even in the absence of prior antibiotic exposure. The limitations of pathogen-focused pneumonia diagnostics underscored by this study, namely the inability to distinguish true infection from incidental pathogen carriage, were a key motivation for our study. We have now referenced this work in our introduction to underscore the longstanding diagnostic challenge:

> Line 66: "Early postmortem studies of direct lung tissue demonstrated poor concordance between lung culture and histologically confirmed pneumonia, underscoring the limitations of microbiology-based studies alone.[5] However, in contemporary clinical practice, detection of any potential pathogen, particularly in the setting of respiratory failure, can reflexively lead to an LRTI diagnosis, even in the absence of true infection[6,7]."

C7. Finally, the authors highlight the appearance of anaerobes in their IPC cohort. Some intensivists "believe" in the role of these organisms as true lung pathogens in the absence of evidence of aspiration and others don't. They might perhaps consider discussing this a bit further – specially since anaerobic LPS if anything antagonises macrophage activation when

compared to “ordinary” LPS.

Response. We thank the reviewer for raising this important point. We agree that the detection of anaerobic taxa in the lower airway raises clinically relevant questions regarding their pathogenic significance. To address this, we have expanded the discussion to explicitly note that many of the taxa enriched in IPC were oral-respiratory anaerobes and to situate this finding within the broader debate regarding the role of anaerobes as true pneumonia pathogens. We now cite two studies regarding the minimal role of anaerobes in VAP (Marik and Careau, 1999, PMID 9925081) and the harms associated with anaerobic coverage in aspiration pneumonia (Bai et al, 2024, PMID 38387648), as well as the IDSA/ATS 2019 Community Acquired Pneumonia treatment guidelines.

> Line 352: “Many of the taxa enriched in IPC were oral-respiratory anaerobes, intersecting with the ongoing debate regarding the role of anaerobes as true pneumonia pathogens[47,48]. In this context, our findings suggest that enrichment of anaerobic taxa in the lower airway more often reflects innocuous presence within a diverse microbial community rather than invasive infection, aligning with clinical pneumonia guidelines that generally do not recommend empiric anaerobic coverage[49].”

C8. Other than these minor points, the authors are to be congratulated for the very considerable clinical and academic relevance of this work. I look forward to the same approach being applied to an adult population as I struggle to consider whether results will be significantly different, or not. Either way, I suspect that this this will also be a very important publication.

Response. We thank the reviewer for their kind and encouraging comments. We agree that extending this approach in adult populations is a key next step, and as noted above in our response to C5, we are planning parallel analyses in adult cohorts.

C9. Finally, the work prompts the possibility of macrophage FABP4 as a differential diagnostic in thsi clinical space. Shooudl this prove clicnially successful, thsi will be a very major advance in the limitation of inapprorpiate antibitois in this setting; such a finding would be an excellent example of successful translation from CLinical Science to Practice.

Response. We thank the reviewer for this comment and agree that FABP4 holds promise as a clinically translatable biomarker in this setting. As noted in our manuscript, these findings build upon prior work demonstrating the utility of FABP4 as a pneumonia biomarker, and we very recently reported additional supporting data combining FABP4 with a large language model to even more accurately diagnose pneumonia (Phan et al., 2025, PMID 41402257). We have now added this study as a reference in the manuscript. Translation of FABP4 onto a practical gene- or protein-based diagnostic assay is an area of ongoing work in our laboratory.

**Reviewer #2:**

C10. In this study, Lydon et al., evaluated the metatranscriptome of tracheal aspirate from 326 mechanically ventilated children with clinically adjudicated LRTI (n=207), IPC (n=70), and non-infectious acute respiratory illnesses (n=49). There are several strengths here. This study tries to address an important problem, where incidental pathogen carriage frequently happens and we do not know how to interpret or what to do. This culture independent method seems to provide a new view on this issue. The use of metatranscriptomic RNA sequencing is a cutting edge unfrequently used technique for lower respiratory tract studies.

Response. We thank the reviewer for the thoughtful comments about the strengths of our study. We agree that incidental pathogen carriage represents a major unresolved challenge in the diagnosis and management of respiratory infections as well as a major gap in the literature. We appreciate the reviewer's recognition of novelty and potential clinical relevance of our integrated host-microbial metatranscriptomics approach.

C11. In abstract: "The single gene FABP4, when combined with alpha diversity, performed similarly, and FABP4 protein alone achieved an AUC=0.88 (95% CI 0.82–0.93).". A mention of what FABP4 would facilitate the reading of the abstract.

Response. We thank the reviewer for this suggestion and agree. We have revised the abstract to briefly describe FABP4:

> Line 47: "The single gene *FABP4*, which encodes a macrophage-associated lipid chaperone and is a recently described pneumonia biomarker, performed similarly when combined with alpha diversity; FABP4 protein alone achieved an AUC=0.88 (95% CI 0.82–0.93)."

C12. A key factor here is the presence or absence of clinical LRTI as described in the methods. One can probably infer what that means but there might be some room for difference of criteria. For example, was this only based on imaging, or changes in oxygeneation patter, fever, leukocytosis, etc., were also considered. They should add this criteria in the methods.

Response: We appreciate the reviewer raising this excellent point, which overlaps with Comment C2 raised by Reviewer 1. We agree that explicit description of the clinical criteria/definition used for adjudicating LRTI versus non-infectious respiratory failure is key for transparency and reproducibility.

As recommended by the reviewer, we have added a new **Supplementary Methods** section that details the clinical adjudication process and the specific features considered by adjudicators. Adjudication was performed retrospectively by ≥2 independent clinicians with expertise in pediatric infectious diseases and/or critical care, and adjudicators were instructed to utilize the Center for Diseases Control (CDC)/National Healthcare Safety Network (NHSN) Pneumonia (PNEU) 1 definition. Specifically, respiratory symptoms, vital signs, physical exam findings, laboratory values (including white blood cell indices and blood gas parameters), and radiology findings are all incorporated in the PNEU1 definition.

C13. In some patients nasopharyngeal swabs were utilized for identification of respiratory pathogen. Those could have nasal incidental pathogen carriage and not lower respiratory tract incidental pathogen carriage. Those are two distinct scenarios. Did the authors try to separate the two?

Response. We appreciate this important and nuanced point raised by the reviewer. We agree that pathogen detection in the upper versus lower respiratory tract represents distinct biologic scenarios, and that upper airway carriage does not necessarily imply lower respiratory tract carriage.

In this study, microbiologic positivity was defined as a composite of standard-of-care clinical testing and a validated method for lower respiratory tract pathogen detection using metatranscriptomics that has been found to accurately identify culture or PCR-confirmed pathogens while enabling additional detection in culture-negative cases of LRTI (Langelier and Kalantar et al., 2018, PMID 30482864; Tsitsiklis et al., 2022, PMID 35544065; Mick et al., 2023, PMID 37009900). Importantly, all clinically identified bacterial pathogens were detected via lower respiratory tract sampling, so this concern applies primarily to the viral pathogens identified by nasopharyngeal respiratory viral panels.

To explore this, we reviewed concordance between upper respiratory clinical testing and lower respiratory tract metatranscriptomics data. Only 9 patients (2.9% of the LRTI cases and 4.3% of the IPC cases) had viral pathogens detected exclusively by nasopharyngeal respiratory viral panels. Notably, in 4 out of 9 of these cases, the same virus was present in the tracheal aspirate sequencing data but fell below our predefined abundance and/or background-filtering thresholds. These data indicate that discordant upper-only viral detection only occurred in a small minority of cases and is unlikely to meaningfully impact our study conclusions. We have added this detail to our new Supplementary Methods section:

> Line 177: “Standard-of-care clinical viral testing was frequently performed on upper respiratory tract specimens, whereas host transcriptomic and microbiome profiling in this study was conducted exclusively on lower respiratory tract samples (tracheal aspirates). Because microbial detection and carriage can differ between upper and lower airway compartments, and because the biological focus of this study was the lower respiratory tract, we specifically reviewed discordance between upper and lower respiratory tract pathogen detection that would have altered microbiologic classification. Such discordance was rare, occurring in only 9 patients (6 LRTI and 3 IPC; 2.9% of LRTI and 4.3% of IPC cases). Notably, in 4 of these 9 cases, the same viral pathogen was detected in lower respiratory tract metatranscriptomic data but fell below predefined abundance and/or background-filtering thresholds. These findings suggest that our microbiologic definitions were robust, reflected lower airway biology, and aligned with how respiratory pathogen detection is interpreted in routine clinical practice.”

For transparency, our clinical microbiology and metatranscriptomic pathogen detection is publicly available in the metadata file of our GitHub repository. In addition, our research group has previously examined concordance between the upper respiratory tract PCR testing and tracheal aspirate metatranscriptomics in this same cohort, demonstrating generally high agreement (Osborne et al, 2024, PMID 37732845).

C14. Line 121: “Antibiotic usage prior to intubation was similar across…” A different an important data is to evaluate antibiotic usage prior to sampling as this could modify the evaluation of the metatranscriptome. This could then be used as a confounder to adjust some analyses.

Response. We appreciate the reviewer raising this important point and agree that prior antibiotic exposure can influence airway microbial communities and metatranscriptomic profiles. In this

cohort, some patients received antibiotics prior to intubation, either in the outpatient setting or during earlier inpatient care. As shown in Table 1, however, antibiotic use prior to intubation was statistically similar across LRTI, IPC, and CTRL groups (approximately 30% in each), indicating that this was unlikely contributing to any confounding of analyses.

Importantly, enrollment and lower respiratory tract sampling occurred within 24 hours of intubation, thereby minimizing prolonged or differential antibiotic exposure immediately preceding sample collection. Given the comparable prevalence of pre-intubation antibiotic use across groups and the short interval between intubation and sampling, we did not include antibiotic exposure as an adjustment variable in primary analyses. We agree that antibiotic timing and duration are critical considerations for longitudinal microbiome studies and note this as an important factor when interpreting cross-sectional airway microbiome data.

C15. Figure 1 shows “Distribution of pathogens among patients with clinically adjudicated lower respiratory tract infection (LRTI) or incidental pathogen carriage (IPC). Bar plot demonstrating the proportion of participants (…) with each detected pathogen as determined by combined clinical testing and metatranscriptomics”. This is bit problematic since there is likely variability of the clinical testing performed across different patients (for example, not every patient might have gotten a respiratory viral panel, or anaerobic cultures, etc.). While this is un avoidable, I would recommend to report the results of clinical testing separately from metatranscriptome.

Response. We thank the reviewer for this helpful suggestion. We added a new **Supplementary Figure S2** that delineates whether each pathogen was identified by clinical testing alone, metatranscriptomics alone, or by both methods. We believe this approach balances readability with detail, allowing interested readers to examine detection modality in greater detail without overcomplicating the main figure. Detailed per-patient clinical microbiology and metatranscriptomic data are also available in our public GitHub repository metadata file, for readers who wish to further explore these relationships.

For ease of reviewing, the new **Figure S2** and caption are shown below:

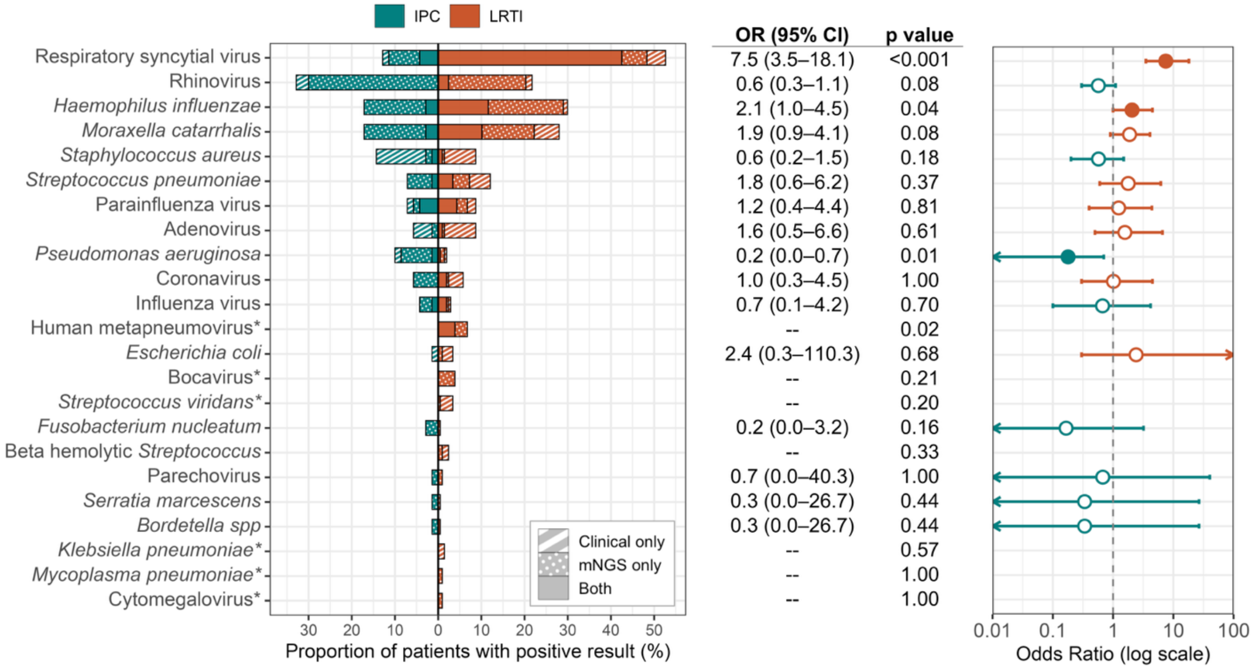

**Figure S2. Pathogen distribution stratified by detection modality in lower respiratory tract infection (LRTI) and incidental pathogen carriage (IPC).** Bar plot demonstrating the proportion of participants in the LRTI (n = 207, orange) and IPC (n = 70, teal) groups with pathogen detection stratified by clinical testing, metatranscriptomics, or both. Bar pattern denotes detection modality. Odds ratio (OR) with 95% confidence interval (CI) tabulated and plotted on the right. Filled circles represent statistically significant pathogens (P<0.05). Asterisks indicate pathogens detected exclusively in one group, for which an OR could not be estimated. Arrows indicate where the lower or upper confidence interval exceeds the plotting area. Pathogens detected only once in the entire cohort were excluded from plotting. Statistical significance for between-group differences was assessed with Fisher's exact test.

C16. There is no data shown on background (technical) control samples which is commonly needed in low biomass studies.

Response. We agree with the reviewer that background and reagent contamination is an important consideration in low-biomass respiratory microbiome samples, particularly when using highly sensitive metatranscriptomic sequencing approaches.

First, we would like to note that all tracheal aspirate samples were processed using a rigorous ultra-clean protocol designed to minimize contamination. This included wearing personal protective equipment (gown, sterile gloves, and face shield) when handling samples, wiping down pipettes and work surfaces with RNaseZap (Ambion) to remove RNase contamination, followed by 70% ethanol for additional disinfection, and maintaining strict physical separation of work areas. Samples were thawed by extraction batch, and all tubes and plates were kept sealed or covered at all times until use. We have clarified these procedures in the Methods section:

> Line 499: "All sample handling and RNA extractions were performed using a rigorous ultra-clean protocol designed for low biomass specimens, including use of personal protective equipment, RNase-decontaminated work surfaces and instruments, strict workflow separation, and processing of water-only controls in parallel to monitor and account for background contamination."

Despite this ultraclean protocol, some degree of contamination is unavoidable. We therefore processed double-distilled water samples as technical controls in parallel with our clinical samples. For this study, we included 34 water controls. We incorporated these background controls into our analytic pipeline in order to estimate taxon-specific background distributions, which are then included in our previously validated negative-binomial model to determine whether a given taxon was present at an abundance above the expected background in each clinical sample. Only taxa exceeding this background threshold were retained for downstream analyses. This is described in further detail in the Methods section "Identification and mitigation of background contaminants".

We have added a new Supplemental Data file (**Supplementary Data File 8**) tabulating the reads (counts based on the NCBI nucleotide database at the species level) in the water control samples that passed our described QC filters and exceeded modeled background contamination thresholds. Of the 24 water control samples processed, only 6 contained taxa exceeding background thresholds.

For reviewers and readers interested in additional detail, the complete background-model output including species level taxonomic hits, full NT/NR alignment metrics, and statistical testing results from the background contamination model for both clinical samples and water controls is publicly available on our project GitHub page (file name: microbial_data_bg_corrected.csv). To further facilitate reviewer evaluation, we have now added a simple CSV file listing the water control sample identifiers (file name: water_sample_names.csv) to our GitHub.

C17. Line 147: "Community composition also differed between LRTI, IPC, and CTRL groups based on the Bray-Curtis index (P<0.001) (Figure 2c)." In the supplement in fig S6 they also report a beta diversity plot comparing these three groups but the distribution is very different. Please clarify.

Response. We appreciate the reviewer's comment and the opportunity to clarify this point. Figure 2c and Figure S7 (previously Figure S6) represent different types of analyses applied to different data modalities.

Figure 2c shows microbiome beta diversity, calculated using Bray-Curtis dissimilarity and visualized by principal-coordinate analysis (PCoA), and therefore reflects differences in lower airway microbial community composition between LRTI, IPC, and CTRL groups.

In contrast, Figure S7 displays principal component analysis (PCA) of the lower airway host transcriptome, showing variation in global host gene expression across samples. These panels were included in the supplement to provide additional views of the host transcriptomic ordination (PC1–PC2 and PC2–PC3), complementing the PC1–PC3 plot shown in the main text (Figure 3a), which was selected because it provided the clearest visual separation between groups.

Because these figures depict different biological data (microbiome versus host response) and use different distance metrics and ordination methods, their visual distributions are not expected to align. To improve clarity, we have updated the Figure S7 legend to more explicitly explain that this reflects host gene expression, not microbiome diversity.

C18. Among the control patients, some might have had a pathogen identified as such based on their criteria on metatranscriptome data. How many patients had that and how does that compare with IPC and LRTI?

Response. We appreciate the reviewer's question. We have added a new figure, **Figure S2**, to explicitly distinguish pathogens detected by clinical microbiology alone, metatranscriptomics alone, or both.

We also appreciate the opportunity to clarify how metatranscriptomic pathogen detection was incorporated into cohort definitions. As noted by the reviewer, metatranscriptomics was part of the microbiologic reference standard used to define groups, together with standard-of-care clinical testing. A validated rules-based model for detecting lower respiratory tract pathogens using metatranscriptomics that accurately identifies culture or PCR-confirmed pathogens while enabling additional detection in culture-negative LRTI cases was utilized in this study (Langelier and Kalantar et al., 2018, PMID 30482864; Tsitsiklis et al., 2022, PMID 35544065; Mick et al., 2023, PMID 37009900). These supportive validation studies are cited in our Methods section.

Patients were classified as:

1. LRTI if clinically adjudicated as LRTI and a pathogen was detected by clinical testing and/or metatranscriptomics;

2. IPC if clinically adjudicated as not having LRTI but a pathogen was detected by clinical testing and/or metatranscriptomics; or
3. CTRL if clinically adjudicated as not having LRTI and no pathogen was detected by either clinical testing or metatranscriptomics.

Accordingly, by definition, control patients had no pathogens detected by either culture or the rules-based model. We have added a new **Supplemental Methods** section to help clarify group definitions. To further enhance transparency, we provide in the GitHub metadata file the pathogen detection status for each participant by clinical testing and metatranscriptomics.

C19. Line 148: "Despite these distinct microbiome archetypes, the detected pathogen was typically the most abundant microbe in the airway microbiome in both LRTI and IPC (Figure S2)". Is the detection of pathogen here based on clinical results or based on metatranscriptome relative abundance. If the later were included then these results are kind of obvious, no?

Response. We thank the reviewer for this thoughtful question and agree that this point warrants clarification. In our study, "detected pathogen" refers to pathogens identified using our combined microbiologic framework, which integrates standard-of-care clinical testing and metatranscriptomic pathogen calling using a previously validated rules-based model, which we have now described more clearly in the Methods/Supplementary Methods (see responses above).

For viruses, metatranscriptomic pathogen detection is based on identifying established respiratory viral pathogens that exceed a conservative abundance threshold (>0.1 reads per million) after background filtering. For bacteria, pathogen calling is more nuanced and does not simply assign the organism with the highest read count as the pathogen. Instead, the rules-based model described above evaluates each microbe's relative abundance and ecological dominance within the respiratory microbiome, and considers whether a taxon is on a reference list of established respiratory pathogens derived from large epidemiological surveillance studies (Jain et al., 2015, PMID 26650159; Jain et al., 2015, PMID 25714161; Iwai et al., 2014, PMID 24752365; Magill et al., 2014, PMID 24670166). These studies are cited in our Methods section.

Notably, the model does not simply select the top-ranked microbe by abundance, rather it identifies the largest abundance gap among the top 15 most abundant species, if one exists. For example, in some cases, more than one organism may be present at a comparably high abundance, each of which is significantly higher in abundance than all remaining detected species. In such cases, the most abundant microbe may be a commensal organism, while the other microbe present at slightly lower abundance may be an established respiratory pathogen. In this circumstance, only the pathogen will be returned by the model, even though both are dominant over all the other microbes detected. As noted above and in our Methods section, this model has been found to accurately identify culture or PCR-confirmed pathogens while enabling additional detection in culture-negative LRTI cases (Langelier and Kalantar et al., 2018, PMID 30482864; Tsitsiklis et al., 2022, PMID 35544065; Mick et al., 2023, PMID 37009900).

We have included Figure S3 (previously Figure S2) to highlight the frequency at which the pathogen identified by our combined clinical-metagenomic framework was also the most abundant microbe in the community; notably the results were similar across both LRTI and IPC groups. We have now clarified the above as follows:

> Line 153: "Despite these distinct microbiome archetypes, the microbe designated as the pathogen by our combined clinical-metagenomic framework was typically, but not

always, the most abundant microbe in the airway microbiome in both LRTI and IPC (Figure S3)."

C20. For Fig 3b I would expect to see some sort of dendogram supporting the clustering.

Response: We thank the reviewer for this suggestion. We agree that displaying the dendrogram helps clarify the hierarchical clustering shown in Figure 3b. We have now added the patient (column) dendrogram above the heatmap to explicitly show the clustering structure:

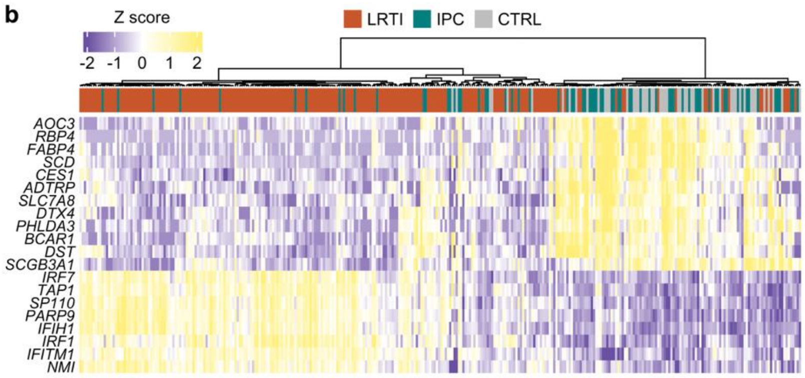


In the original version, the dendrogram was omitted to maximize readability and preserve space in this highly multi-paneled figure. Of note, we describe the clustering methodology in the Methods section "Heat map and hierarchical clustering".

C21. Line 208: "Among the genes DE between LRTI and both CTRL and IPC groups, we noted that FABP4, which is expressed in macrophages and encodes a lipid chaperone that modulates leukotriene stability38, was a clear outlier in both fold change and statistical significance (Figure 3f)." What criteria supports that FABP4 is a clear outlier (or extreme value). This could be shown by indicating where this gene fall within the volcano plot.

Response: We thank the reviewer for this helpful suggestion. To explicitly demonstrate that *FABP4* is an extreme outlier in both effect size and statistical significance, we have added a new Supplementary Figure (**Figure S8**) showing the same volcano plots as in Figure 3c (LRTI vs CTRL) and Figure 3d (LRTI vs IPC), with *FABP4* labeled.

As demonstrated in this new figure, *FABP4* lies among the most highly significant and most strongly differentially expressed genes in both comparisons, supporting its designation as a clear outlier.

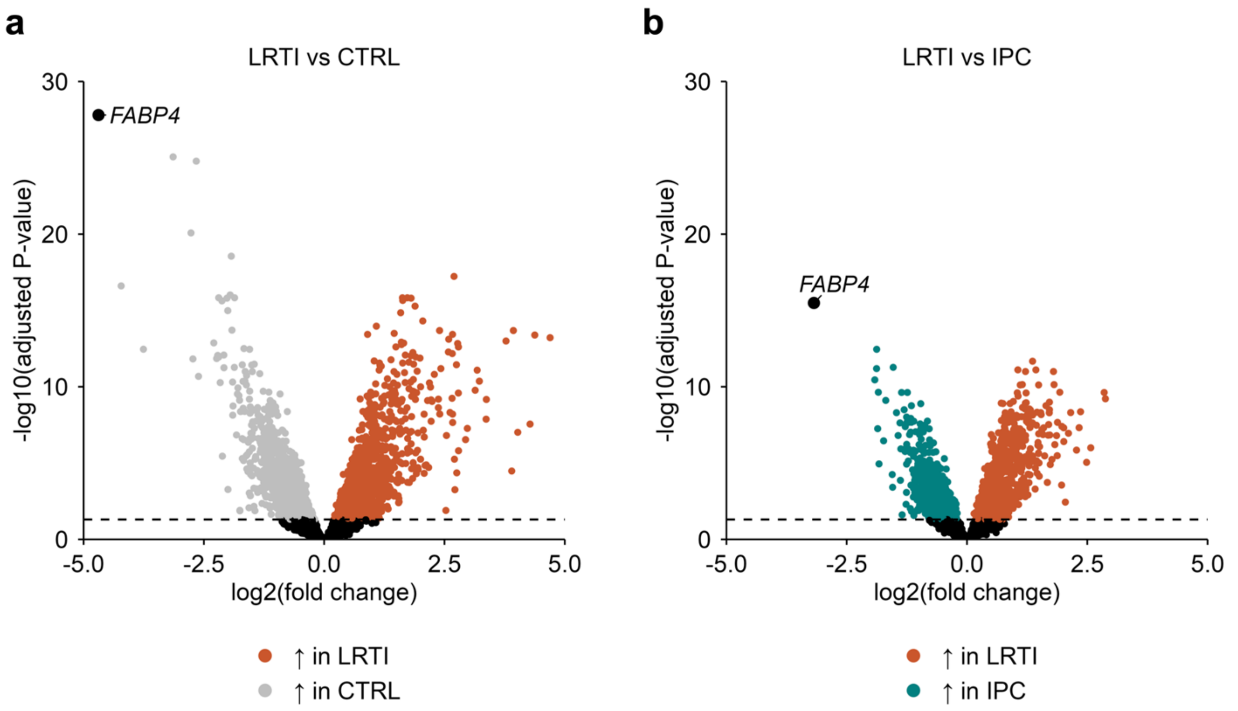


**Figure S8. *FABP4* is an outlier in differential expression analyses comparing LRTI to CTRL or IPC groups.** Volcano plots showing differential gene expression between a. LRTI and CTRL and b. LRTI and IPC groups. Each point represents a gene, plotted by $\log_2$(fold change) and $-\log_{10}$(Benjamini-Hochberg adjusted P value). Genes significantly upregulated in LRTI (orange), CTRL (gray), or IPC (teal) are highlighted. The gene *FABP4* is labeled and shown as a marked outlier in both comparisons, with among the largest effect sizes and highest statistical significance relative to other differentially expressed genes.

C22. Line 337: "Unexpectedly, total bacterial abundance was greater in IPC than in LRTI or controls, which may be explained by the enrichment of commensal taxa such as Prevotella, Neisseria, and Porphyromonas and reflect a more resilient and balanced barrier microbiota." A quantitative method such as ddPCR with a clear target (e.g., 16S rRNA gene) would be more desirable to address this issue.

Response: We agree with the reviewer that absolute quantification of bacterial mass using 16S rRNA ddPCR would be a preferable approach for assessing total bacterial load. Such measurements unfortunately are unavailable. We agree that future studies incorporating orthogonal approaches, such as DNA-based absolute quantification (e.g., 16S PCR) or the use of external RNA spike-in mass standards, would allow for absolute quantification and the ability to truly assess differences in bacterial mass/burden between groups. We have added this as a future direction in our Discussion section.

> Line 436: "Our metatranscriptomic measurements reflect relative microbial abundance rather than absolute microbial mass or burden; future studies incorporating more quantitative orthogonal approaches (e.g., 16S rRNA gene quantification or spike-in mass standards[66] will be useful to more precisely estimate microbial burden.

Minor:
C23. In Abstract: “Together, our findings reveal distinct ecological and immunologic archetypes that define LRTI and IPC, and support data-driven, biology-informed LRTI diagnostics that incorporate host and microbe.” Maybe “that incorporate host and microbe signatures” or “biomarkers”

Response: We thank the reviewer for this helpful suggestion. We agree that specifying the nature of what is incorporated improves clarity. We have revised the wording as follows:

> Line 50: “Together, our findings reveal distinct ecological and immunologic archetypes that define LRTI and IPC, and support data-driven, biology-informed LRTI diagnostics that incorporate host and microbial features.”

C24. Table 1 should be referenced in the text, probably in the third paragraph of the results.

Response. We thank the reviewer for noting this omission. We have now added a citation to Table 1 in the third paragraph of the Results section, where the baseline clinical and demographic characteristics of the cohort are described.

C25. (Remarks on code availability): seems appropriate thought I did not try to run it

Response. We thank the reviewer for taking the time to review our code and data on GitHub.
